# MMP7-Triggered mitophagy by regulating parkin-mediated ubiquitination of VDAC1 confers anoikis resistance in the epithelium of hyperplastic prostate

**DOI:** 10.1038/s41419-026-08867-y

**Published:** 2026-06-01

**Authors:** Yongying Zhou, Yan Li, Huan Liu, Junchao Zhang, Jizhang Qiu, Lu Du, Huan Lai, Ping Chen, Yuming Guo, Yongwen Luo, Michael E. DiSanto, Xinhua Zhang

**Affiliations:** 1 Department of Urology, Zhongnan Hospital of Wuhan University, Wuhan University, Wuhan, China; 2 Department of Urology, the Affiliated LiHuiLi Hospital of Ningbo University, Ningbo University, Ningbo, China; 3 Department of Urology, Xijing Hospital of Fourth Military Medical University, Xi’an, China; 4 Department of Surgery and Biomedical Sciences, Cooper Medical School of Rowan University, Camden, NJ USA

**Keywords:** Apoptosis, Prostatic diseases

## Abstract

Benign prostatic hyperplasia (BPH), prevalently in aging men, is characterized by aberrant cell death of prostate cells. Anoikis, a specific subtype of apoptosis, is triggered when cells detach from the extracellular matrix (ECM), in contrast, cells with anoikis resistance contribute to pathological processes such as unregulated cell proliferation and impaired cell death. However, the role of anoikis resistance in BPH pathogenesis remains poorly understood. In this study, an elevated anoikis resistance level was observed in BPH tissues compared to normal prostates. Furthermore, matrix metalloproteinase 7 (MMP7) was identified as a key regulator of anoikis resistance in hyperplastic prostatic epithelium. Under anoikis-inducing conditions, MMP7 promoted mitophagy via the PINK1-Parkin pathway, alleviated mitochondrial stress damage, and enhanced anoikis resistance in BPH-1 cells. Mechanistically, MMP7 interacted with VDAC1 and bound specifically to lysine residues K109 and K110, thereby inhibiting VDAC1 oligomerization and increasing the accumulation of VDAC1 monomers, which served as additional binding sites to Parkin-mediated polyubiquitination. Moreover, clinical data revealed that MMP7 expression levels correlated significantly with the International Prostate Symptom Score (IPSS) and nocturia frequency. Additionally, in vivo experiments demonstrated that inhibition of MMP7 suppressed mitophagy and markedly attenuated prostatic epithelial hyperplasia in a rat model BPH. Collectively, our findings clarify the functional role of the MMP7-VDAC1 axis in BPH pathogenesis and highlight its potential as a therapeutic target for BPH management.

## Introduction

Benign prostatic hyperplasia (BPH) is a common urological disorder among elderly men, with an age-dependent incidence of up to 90% in men over the age of 85 years [1]. Patients with BPH are predisposition to lower urinary tract symptoms (LUTS), which significantly impairs their quality of life and exert a heavy burden on healthcare resources [2]. Although laboratory models have demonstrated that androgens and growth factors can stimulate the proliferation of prostate cells, there is no conclusive evidence indicating the existence of an active proliferative process in the prostates of middle-aged and elderly men [3–5]. Therefore, although enhanced cellular proliferative activity may contribute to the development of benign prostatic hyperplasia, its importance and specific mechanisms in the initiation and progression of the disease remain unclear. Apoptosis, one of the earliest identified and most extensively studied forms of PCD, is widely accepted to be reduced in BPH [6–8]. Notably, B-cell lymphoma 2 protein (Bcl-2)—a well-recognized canonical member of the anti-apoptotic protein family—has been documented to be upregulated in both basal and secretory epithelial cells of BPH tissues [9]. In line with this observation, our group’s previous investigations have further validated a pronounced upregulation of Bcl-2 protein expression in the prostate tissues of BPH rats, which suggests a predisposition to attenuated prostate apoptosis during BPH pathogenesis [10]. Nevertheless, to date, the overwhelming majority of PCD-centric studies have focused on cancer progression, whereas research addressing the mechanistic link between PCD dysregulation and BPH remains remarkably limited. Thus, it is of great necessity to delve into the molecular mechanisms of the dysregulated PCD which could lead to the onset and progression of BPH.

Anoikis, is defined as a special form of PCD that occurs when cells detach from adjacent cells or the extracellular matrix (ECM) [11, 12]. Anoikis plays a critical role in the context of human epithelial cells [13, 14]. During embryonic development, anoikis of epithelial cells facilitates the lumen formation of intestine and acini of glands, including the mammary and prostate glands [15]. Physiologically, anoikis functions to eliminate detached epithelial cells, contributing to the homeostasis of glandular tissue [16]. Mechanistically, anoikis relies on the mitochondrial-mediated intrinsic pathway and death receptor-mediated extrinsic signaling. The intrinsic pathway is induced by the upregulation of pro-apoptotic molecules (such as BAX, Bad, Bik, and Puma), which inhibits the anti-apoptotic protein Bcl-2 family and mediates the Caspase 9-Caspase 3 cascade. The extrinsic pathway is initiated by increasing the expression of Fas receptors, triggering the Caspase 7-Caspase 3 cascade, or through truncated Bid (tBid) to promote the release of mitochondrial cytochrome c and the assembly of apoptotic bodies [17–19]. Under pathological conditions, detached cells are capable of resisting anoikis for survival, resulting in an impaired regulation of PCD, which is designated as anoikis resistance [20, 21]. Notably, as early as 1989, Isaacs and Coffey demonstrated that prostate glandular epithelial cells detached from the stroma exhibited characteristics such as resistance to apoptosis, loss of secretory function, and excessive proliferation. This phenomenon was termed the “Brake” theory in the context of BPH progression, and it bears similarity to anoikis resistance [22]. However, the exact role of anoikis resistance in the development and progression of BPH remains unclear to date.

To investigate the detailed molecular mechanisms of anoikis resistance related to BPH, we utilized RNA-seq datasets of human enlarged prostates and combined them with RNA-seq datasets of human hyperplastic prostate cells (BPH-1) in an anoikic environment for cross-analysis. Finally, screening identified 7 genes as potential mediators of anoikis resistance in BPH. Among them, matrix metalloproteinase 7 (MMP7) exhibited the most potent regulatory effect on the apoptosis of BPH-1 cells in an anoikis condition. As one of the members in the MMP family, MMP7 is comprised of only a leader peptide along with a catalytic domain, exhibiting unique substrate selectivity due to the absence of a C-terminal domain [23, 24]. Fundamentally, MMP7 can degrade ECM components such as laminin and fibronectin and contribute to embryonic development and tissue remodeling across various diseases. Of note, it is predominantly expressed in glandular epithelial cells (such as those in the breast, pancreas, intestine, and endometrium) playing a role the maintenance of mucosal barrier homeostasis and glandular secretion functions [25–28]. Under pathological conditions, the expression of MMP7 is significantly upregulated and is attributed to diseases such as tumors, fibrosis, and inflammation. Currently, there is no existing evidence that MMP7 modulates anoikis resistance in the development of BPH.

Our GO and KEGG analyses of the RNA-seq data also disclosed that MMP7 was enriched in multiple mitochondrial-related signaling pathways in anoikic condition. As we known, mitophagy selectively eliminates damaged mitochondria, which constitutes a core mechanism for maintaining cellular homeostasis [29, 30]. The initiation of mitophagy depends on two primary signaling pathways. One is the PINK1-Parkin-dependent pathway, where PINK1 accumulates on the outer mitochondrial membrane and recruits Parkin to ubiquitinate outer membrane proteins, such as voltage-dependent anion channel 1 (VDAC1) and MFN2, which are then guided by autophagy receptors to be enveloped by autophagosomes [31, 32]. Another is the receptor-mediated non-ubiquitination pathway, where mitochondrial proteins such as BNIP3 and NIX/FUNDC1 directly bind to LC3 under hypoxia or oxidative stress, bypassing the ubiquitination step to trigger autophagy [33]. This pathway is commonly observed in specific physiological and pathological scenarios such as red blood cell maturation and myocardial ischemia-reperfusion injury [34]. Here, our mass spectrometry analysis demonstrated that MMP7 directly interacts with VDAC1. This interaction suppresses VDAC1 homo-oligomerization, thereby promoting Parkin-mediated polyubiquitination of VDAC1 and subsequent activation of mitophagy.

The current study identified the presence of anoikis resistance in BPH and that MMP7 was systematically invovled as a major regulator of anoikis resistance. MMP7 could impede the homo-oligomerization of VDAC1, while increasing the poly-ubiquitination of VDAC1 by Parkin and activating mitophagy, thereby antagonizing anoikis and facilitating BPH progression. Finally, we verified the role of MMP7 in vivo using a testosterone propionate-induced BPH (TP-BPH) rat model.

## Results

### Measurement of anoikis resistance levels in the BPH prostate tissues

**Fig. 1:**
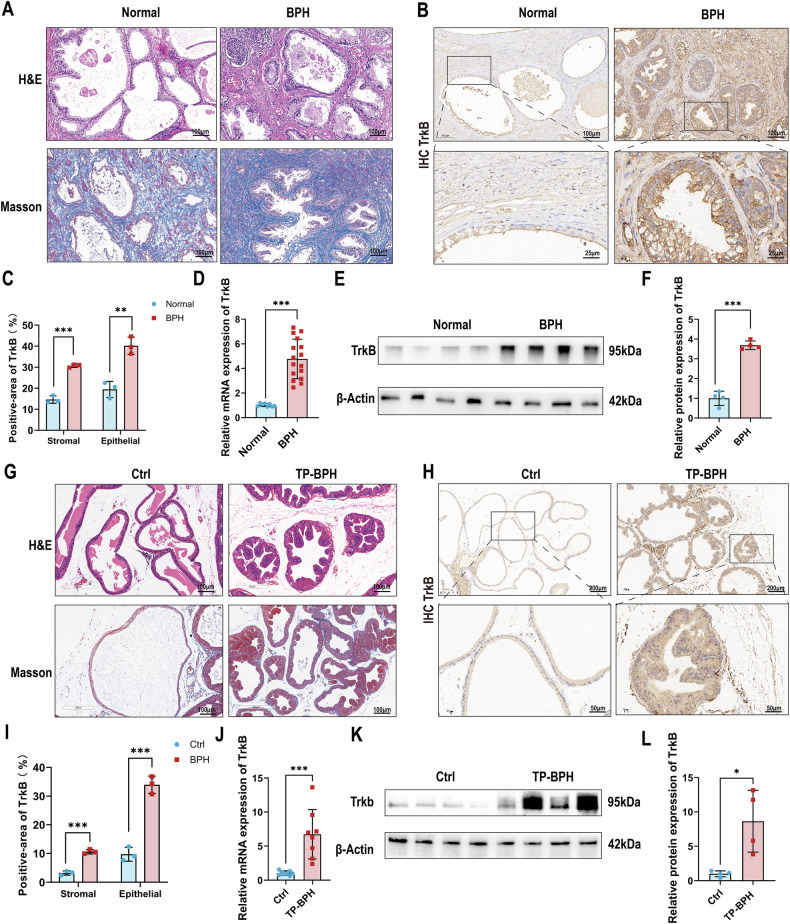
The expression of TrkB in normal and hyperplastic prostate tissues in humans and rats. **A** Representative images of H & E-stained and Masson-stained human prostate tissue specimens from the normal and BPH groups. Scale bars: 100 µm. **B** Representative IHC images showing in-situ TrkB expression in human prostate tissues. Scale bars: 50 µm and 200 µm. **C** Statistical analysis to compare the difference of TrkB-positive area between the normal (*n* = 3) and BPH groups (*n* = 3) using Image J software. **D** qRT-PCR analysis demonstrating the expression level of TrkB gene in normal prostate tissues (*n* = 8) and BPH tissues (*n* = 16). The expression of TrkB was increased in BPH at the mRNA level. **E** Immunoblot assay revealed the protein expression of TrkB in normal prostate tissues (*n* = 4) and BPH tissues (*n* = 4). **F** Statistical graph of Western blot bands showing that the protein expression of TrkB is increased in BPH tissues. **G** Representative images of H & E-stained and Masson-stained rat prostate tissue specimens from the control and TP-BPH groups. Scale bars: 100 µm. **H** Representative IHC images showing in-situ TrkB expression in rat prostate tissues. Scale bars: 50 µm and 200 µm. **I** Statistical analysis to compare the difference of TrkB-positive area between the control (*n* = 3) and TP-BPH groups (*n* = 3) using ImageJ software. **J** qRT-PCR analysis demonstrating the expression level of MMP7 gene in rat prostate tissues from the control (*n* = 8) and TP-BPH groups (*n* = 8), and the expression of TrkB was increased TP-BPH groups at mRNA level. **K** Immunoblot assay revealing the protein expression of TrkB in rat prostate tissue from the control (*n* = 4) and TP-BPH groups (*n* = 4). **L** Statistical graph of Western blot bands showing that the protein expression of TrkB is increased in TP-BPH groups. *p < 0.05, ** *p* < 0.01, *** *p* < 0.001. The data are presented as the means ± SDs. All statistical significance was determined by two-tailed unpaired Student’s t-test.

Human normal and hyperplastic prostate tissues were obtained from our institution and subjected to histological analysis. Specimens from BPH patients exhibited markedly thickened epithelial layers and increased collagen fiber deposition in the stroma (Fig. 1A). Furthermore, IHC staining demonstrated that TrkB, a representative indicator of anoikis resistance, was more highly expressed in BPH tissues than in the control group (Fig. 1B, C). Total RNA and protein were extracted from bulk peripheral zone tissues of clinically collected human normal prostate and BPH specimens (not cell-sorted samples), as well as ventral lobe tissues of rat prostates obtained from in vivo experiments. This observation was corroborated by quantitative analyses of both mRNA and protein levels, which consistently showed the upregulation of TrkB in BPH tissues compared to normal prostate tissues (Fig. 1D, F). We further examined anoikis resistance levels in TP-BPH rat models. As shown in Fig. 1G, induction of TP resulted in an enlarged epithelial compartment while barely affecting the stroma (Fig. 1G). Similar to human tissues, TP-BPH rats exhibited increased TrKB levels, as evidenced by immunoblotting, qRT-PCR and Western blot analyses (Fig. 1H–L).

### Establishment of anoikis resistance cell model and identification of anoikis resistance related core gene MMP7

**Fig. 2:**
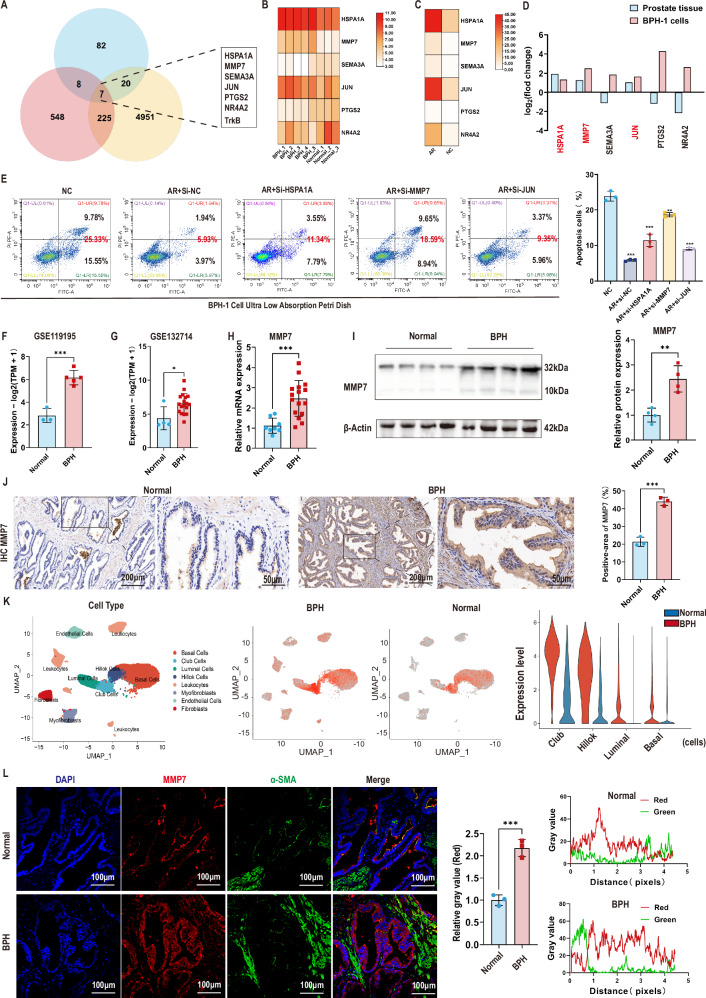
The localization and expression of AR related core gene MMP7 in human prostate tissues. **A** The intersection of differentially expressed gene sets derived from human normal and hyperplastic prostate tissues, BPH-1 and AR-BPH-1 cells, and AR-related genes from the GeneCards database identified a total of seven overlapping genes: HSPA1A, MMP7, SEMA3A, JUN, PTGS2, NR4A2, and TrkB. **B** The heatmap illustrates the expression profiles of 6 differentially expressed genes across human normal prostate tissue samples (*n* = 3) and BPH samples (*n* = 5) (data sourced from the GSE119195 dataset). The color legend on the right depicts the correlation between gene expression levels and the corresponding color intensities. **C** Heatmap of 6 differentially expressed genes in BPH-1 and AR-BPH1 cells by high-throughput transcriptome sequencing. The color scale bar on the right indicates the relationship between the expression value and the color. **D** The log2 (fold change) values of HSPA1A, MMP7, SEMA3A, JUN, PTGS2, and NR4A2 in human prostate tissues (BPH/normal) and BPH-1 cell models (AR-BPH-1 vs. normal BPH-1), only HSPA1A, MMP7, and JUN were upregulated in both BPH tissues and AR-BPH-1 cells. **E** Flow cytometry results and apoptosis level statistics of BPH-1 cells after 24 h culture in an anchorage-independent environment following knockdown of HSPA1A, MMP7, and JUN. **F** The mRNA expression levels of MMP7 in human normal prostate tissues (*n* = 3) and BPH tissues(*n* = 5) based on the GSE119195 dataset. **G** The mRNA expression levels of MMP7 in human normal prostate tissues (*n* = 4) and BPH tissues(*n* = 18) based on the GSE132714 dataset. **H** qRT-PCR analysis demonstrating the expression level of MMP7 gene in normal prostate tissues (*n* = 8) and BPH tissues (*n* = 16), and the expression of MMP7 was increased in BPH at mRNA level. **I** Immunoblot assay revealed the protein expression of MMP7 in normal prostate tissues (*n* = 4) and BPH tissues (*n* = 4), and statistical graph of Western blot bands showing that the protein expression of MMP7 is increased in BPH tissues. **J** Representative IHC images showing in-situ MMP7 expression in human prostate tissues. Scale bars: 50 µm and 200 µm, and statistical analysis to compare the difference of MMP7-positive area between the normal (*n* = 3) and BPH groups (*n* = 3) using Image J softare. **K** UMAP projection of scRNA-seq data from normal prostate and BPH tissues. MMP7 was abundant within epithelial cell clusters including club, hillok, luminal, and basal cells and relatively elevated in these cells from BPH tissues compared to normal. **L** Immunofluorescence localization of MMP7 for normal prostate tissues and BPH tissues, and MMP7 was predominantly localized in epithelium. DAPI (blue) showed nuclear staining; Cy3-immunofluorescence (red) showed MMP7 protein；Alexa fluor 488 (Green) showed α-SMA protein. And negative control in each group omitting the primary antibody failed to stain. Sections of 3 hyperplastic and 3 normal human prostate samples were used for immunofluorescence experiments and cell immunofluorescence staining was repeated at least 3 times. And representative statistical graphs were selected for figures. GAPDH is used as loading control. The scale bars are 100 μm. *p < 0.05, ** *p* < 0.01, *** *p* < 0.001. The data are presented as the means ± SDs. Statistical significance was determined by two-tailed unpaired Student’s t-test **F, G, H, I, J, L** or determined by one-way ANOVA with Tukey’s multiple comparisons **E**.

To establish an anoikis resistancecell model, WPMY-1, RWPE-1, and BPH-1 cells were cultured under anoikis conditions in 10 cm ultra-low attachment culture dishes for 3 days, respectively. The anoikis conditions were established using ultra-low attachment culture dishes, which significantly reduce cell adhesion, protein adsorption, and enzyme activation. This ensures that cells remain non-adherent and in a continuously suspended state throughout the culture process. As shown in Fig. S1A, BPH-1, RWPE-1, and WPMY-1 cells all exhibited normal growth in conventional culture dishes. BPH-1 cells retained superior cellular morphology to RWPE-1 and WPMY-1 cells in anoikis condition. Furthermore, flow cytometry analysis revealed that BPH-1 cells exhibited the strongest anoikis resistance capacity with a 29.96% apoptosis rate, while WPMY-1 and RWPE-1 cells showed significantly weaker anoikis resistance capacity, with apoptosis rates of 61.03% and 43.64%, respectively. Under the condition of normal adherent culture, the apoptosis rates of the three types of cells were all below 5% (Fig. S1B, C). Accordingly, BPH-1 cells were identified as the optimal candidate for the following anoikis resistance studies. BPH-1 cells were further seeded into 10 cm ultra-low adsorption culture dishes, with cell viability and death staining performed at 24, 72 and 120 h (Fig. S1D). Indeed, a large number of BPH-1 cells remained viable in an anoikis condition for 5 days (Fig. S1E). Cells that could reattach when reseeded into normal 10 cm culture dishes were designated as anoikis resistance-BPH-1 cells. Subsequently, transcriptome data from high-throughput RNA sequencing of anoikis resistance-BPH-1 and BPH-1 cells were intersected with dataset of human prostate tissues from GEO database (GSE119195) and anoikis resistance-related dataset from Genecards database, eventually yielding seven differentially expressed anoikis resistance-associated genes: *HSPA1A, MMP7*, *SEMA3A*, *JUN*, *PTGS2*, *NR4A2*, and *TrkB* (Fig. 2A–C). *TrkB* was excluded due to its role as an anoikis resistance marker in multiple diseases. Among the six other genes, only *HSPA1A*, *MMP7*, and *JUN* were upregulated in both BPH tissues and anoikis resistance-BPH-1 cells (Fig. 2D). Additionally, knockdown of HSPA1A, MMP7, and JUN was examined in anoikis resistance-BPH-1 cells, respectively (Fig. S2). The most efficient siRNAs for each gene were selected for further experiments. Furthermore, flow cytometry was employed to assess apoptotic levels of anoikis resistance-BPH-1 cells following reduction of three target genes in anoikis condition for 24 h. Notably, suppression of MMP7 elicited the most pronounced elevation of apoptosis, indicating its dominant regulatory role in anoikis resistance (Fig. 2E). Moreover, transcriptomic analysis of dataset GSE119195 and GSE132714 demonstrated that MMP7 was significantly upregulated in human BPH tissues compared with normal ones (Fig. 2F, G). Parallelly, the mRNA expression and protein level of MMP7 was evaluated in human normal and hyperplastic prostate specimens obtained from our institute, substantiating consistent elevation of MMP7 in BPH tissues (Fig. 2H–J). To further determine the localization of MMP7, single-cell dataset GSE145928 was analyzed to elucidate the expression level of MMP7 across cell types from the BPH transition zone and normal prostate tissues, revealing that MMP7 was abundant within epithelial cell clusters including club, hillok, luminal, and basal cells and relatively elevated in these cells from BPH tissues compared to normal ones (Fig. 2K). Additionally, tissue immunofluorescence staining of human prostate tissues showed that MMP7 was predominantly expressed in the epithelial component, evidenced by absence of co-localization with the stromal marker α-SMA, thus confirming its epithelial-specific expression (Fig. 2L).

### MMP7 regulated mitochondrial metabolism and morphology of anoikis resistance-BPH-1 Cells

**Fig. 3:**
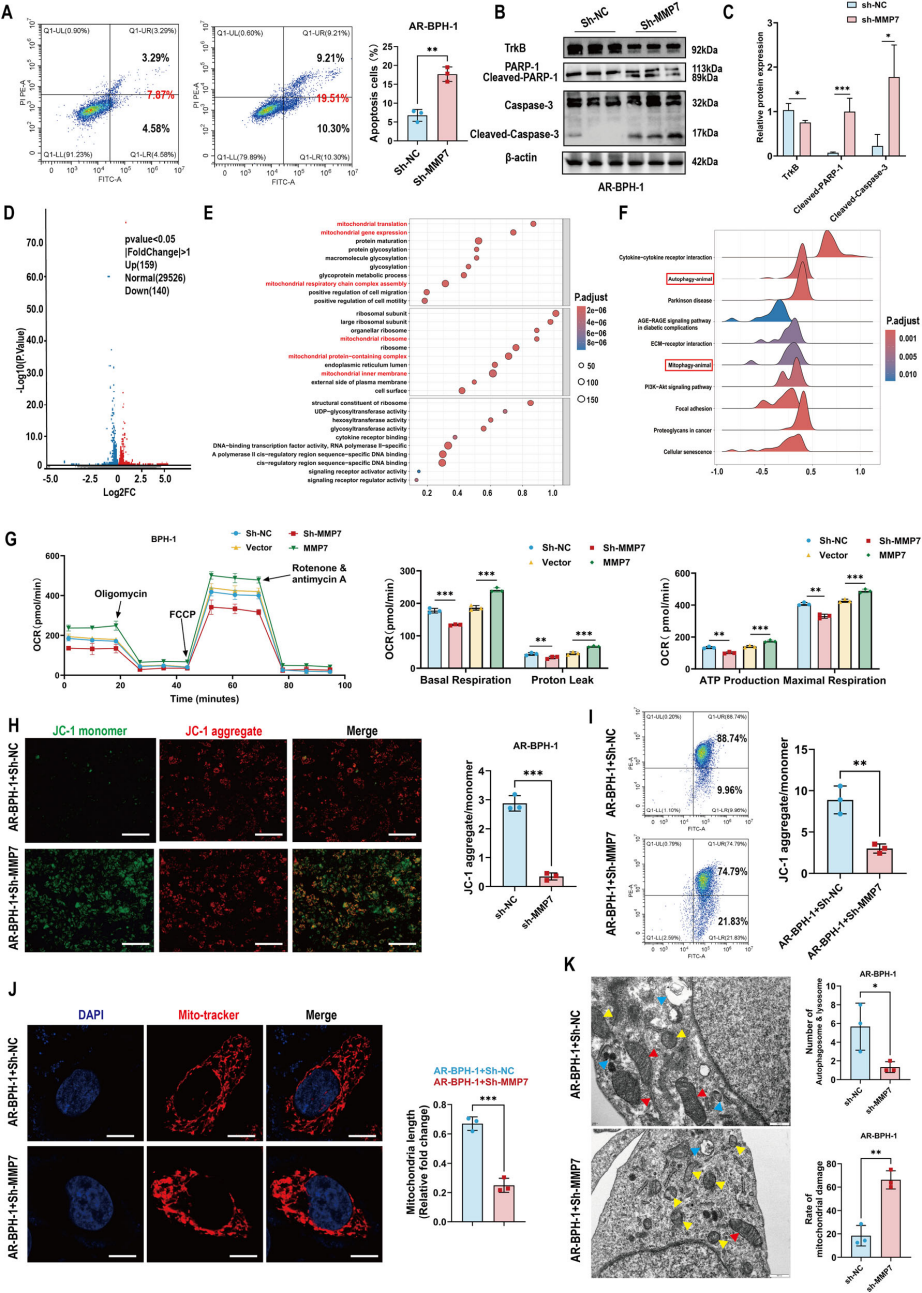
MMP7 regulating mitochondrial metabolism and morphology in AR-BPH-1 cells. **A** Representative flow cytometry images and statistical analysis of apoptosis levels in sh-NC-AR-BPH-1 (*n* = 3) and sh-MMP7-AR-BPH-1 (*n* = 3) cells following 24-h culture in an anchorage-independent environment. **B** Representative Western blot bands showing the effects of MMP7 knockdown in AR-BPH-1 cells on the protein expression levels of TrkB, PARP-1, and Caspase-3. **C** Statistical graph of Western blot bands showing the protein expression of TrkB, PARP-1, and Caspase-3 in AR-BPH-1 cells (*n* = 3) following MMP7 knockdown. **D** Volcano plots illustrating the global gene expression profiles of RNA-Seq analysis. Red dots indicate up-regulated genes (*n* = 159), blue dots indicate down-regulated genes (*n* = 140), and black dots represent genes with no significant change in expression (*n* = 29526). **E** GO analysis for RNA-Seq. Mitochondrial-associated clusters are marked by red. **F** Top 10 enriched pathways from KEGG of RNA-Seq. Autophagy-animal and Mitophagy-animal are shown in red. **G** Oxygen consumption rate (OCR) alterations in sh-NC, sh-MMP7, vector and overexpression MMP7 in BPH-1 cells (*n* = 3). **H** JC-1 staining was employed to assess the mitochondrial membrane potential in sh-NC-AR-BPH-1, and sh-MMP7-AR-BPH-1 cells (*n* = 3). 585/590 nm immunofluorescence (red) showed JC1 aggregates, 514/529 nm immunofluorescence (green) showed JC1 monomers. The scale bars 200 μm. **I** Flow cytometry analysis revealed the distribution patterns of JC-1 aggregates (Q1) and monomers (Q4) across the cell populations. **J, K** The sh-NC-AR-BPH-1 and sh-MMP7-AR-BPH-1 cells were cultured under anchorage-independent conditions for 48 h, followed by collection and fixation, and finally subject to observation by transmission electron microscopy. The ultrastructure of the mitochondria and autophagosome was captured. The red arrows indicated well-preserved mitochondria exhibiting relatively complete mitochondrial cristae and an intact outer membrane, with a long spindle-like morphology; the yellow arrows indicated mitochondria with nearly absent cristae and, in some cases, a ruptured outer membrane, displaying a rounded morphology; the blue arrows indicated autophagosomes. The scale bars are 500 nm. **K** Representative immunofluorescence analysis of Mitochondria length in sh-NC-AR-BPH-1 and sh-MMP7-AR-BPH-1 cells (*n* = 3). DAPI (blue) showed nuclear staining; Cy3-immunofluorescence (red) showed mitochondrion. The scale bars 20 μm. The corresponding statistical analysis of gray-value was presented on the left. The analysis of mitochondrial length was performed using the “Mitochondrial Analyzer” plugin in Fiji software. ns > 0.05, *p < 0.05, ** *p* < 0.01, *** *p* < 0.001. The data are presented as the means ± SDs. Statistical significance was determined by two-tailed unpaired Student’s t-test **A, B, H, I, J, K** or determined by one-way ANOVA with Tukey’s multiple comparisons **G**.

Lentiviral-mediated delivery of MMP7-targeting shRNA was used for gene knockdown. All three MMP7—targeting shRNA constructs (shMMP7-1, shMMP7-2, shMMP7-3) significantly reduced the mRNA and protein levels of MMP7, with shMMP7-3 (sh-MMP7) exhibiting maximal knockdown efficiency, thus being utilized for subsequent assays (Fig. S3). Flow cytometry assay showed that inhibition of MMP7 resulted in a noticeable increase of apoptotic anoikis resistance-BPH-1 cells after 24 h of culture in anoikis condition (Fig. 3A). Similarly, Western blot analysis demonstrated a substantial reduction of TrkB level, and the expression levels of cleaved PARP and cleaved caspase-3 were markedly elevated, indicating enhanced apoptotic activity after MMP7 knockdown (Fig. 3B & C). To further explore the functional pathway, anoikis resistance-BPH-1-sh-MMP7 cells and control anoikis resistance-BPH-1-sh-NC cells were subjected to RNA sequencing analysis, revealing 159 upregulated and 140 downregulated genes in the context of MMP7 knockdown (Fig. 3D). Additionally, GO functional enrichment analysis indicated that MMP7-associated genes were considerably abundant in mitochondrial-related pathways, including “mitochondrial translation”, “mitochondrial gene expression”, “mitochondrial respiratory chain complex assembly”, “mitochondrial ribosome”, “mitochondrial protein complex”, and “mitochondrial inner membrane” (Fig. 3E). Furthermore, gene set enrichment analysis (GSEA) revealed that the deficiency of MMP7 appreciably suppressed autophagy and mitophagy-related pathways, suggesting its critical role in orchestrating mitophagy (Fig. 3F). To verify that MMP7 could affect mitochondrial metabolism in BPH-1 cells, Seahorse experiments were performed to measure the oxidative phosphorylation rate. As shown in Fig. 3G, the oxygen consumption rate (OCR) of anoikis resistance-BPH-1 cells was significantly higher than that of control BPH-1 cells, while MMP7 knockdown effectively reduced the OCR of BPH-1 cells and MMP7 overexpression increased the OCR of BPH-1 cells, indicating that MMP7 regulated oxidative metabolism in anoikis resistance-BPH-1 cells. Mitochondrial membrane potential assay (JC-1 fluorescence probe method) revealed, through immunofluorescence staining (Fig. 3H & I) and flow cytometry respectively, that the loss of MMP7 significantly attenuated Δψm. We also examined mitochondrial morphology using confocal microscopy. As shown in Fig. 3K, anoikis resistance-BPH-1 cells exhibited abundant normal and elongated mitochondria, whereas MMP7 depletion resulted in a shift toward short rod-like and granular mitochondrial structures (Fig. 3J). Furthermore, transmission electron microscopy confirmed that MMP knockdown was associated with reduced or absent mitochondrial cristae, rupture of the outer mitochondrial membrane, and a marked reduction in the number of autophagosomes and lysosomes (Fig. 3K).

### MMP7 modulated mitophagy of anoikis resistance-BPH-1 Cells

**Fig. 4:**
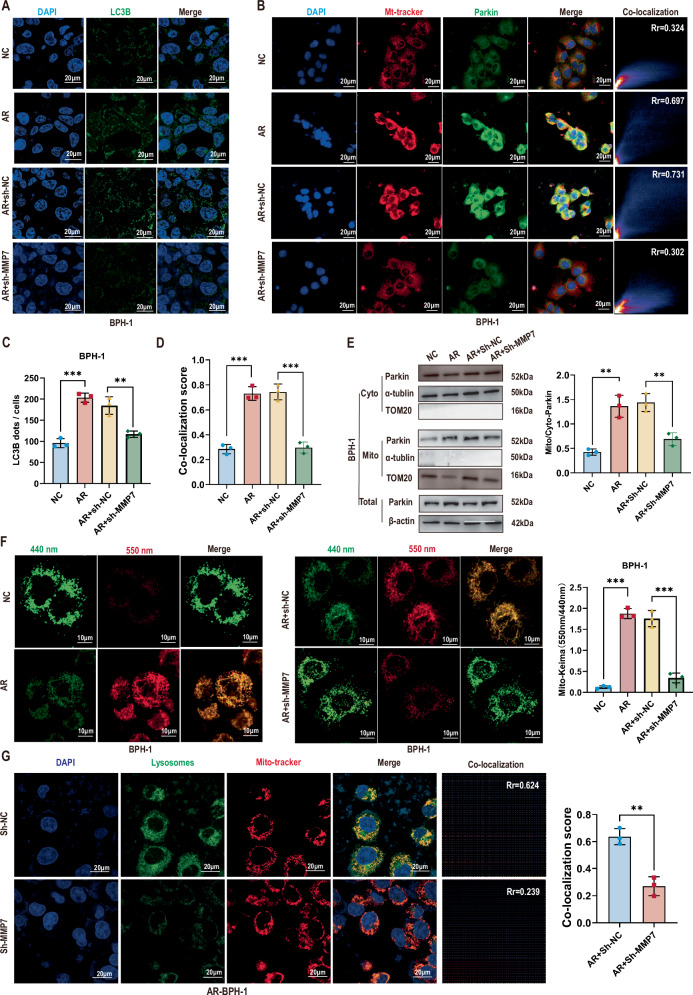
MMP7 was required for mitophagy in AR-BPH-1 cells. **A** Representative images of LC3B immunofluorescence staining in BPH-1, AR-BPH-1, sh-NC-AR-BPH-1 and sh-MMP7-AR-BPH-1 cells. DAPI (blue) shows nuclear staining; Alexa fluor 488 (Green) shows LC3B protein. The scale bars are 20 μm. **B** Representative images of the co-localization staining of Parkin and Mito-tracker in BPH-1, AR-BPH-1, sh-NC-AR-BPH-1 and sh-MMP7-AR-BPH-1 cells. DAPI (blue) shows nuclear staining; Alexa fluor 488 (Green) showed Parkin protein. Cy3-immunofluorescence (Red) showed Mito-tracker. The scale bars are 20 μm. **C** Quantification of LC3B immunofluorescence staining in BPH-1, AR-BPH-1, sh-NC-AR-BPH-1 and sh-MMP7-AR-BPH-1 cells. **D** Quantification of the co-localization staining of Parkin and Mtio-tracker in BPH-1, AR-BPH-1, sh-NC-AR-BPH-1 and sh-MMP7-AR-BPH-1 cells. **E** Representative Western blot bands show the accumulation of Parkin protein on mitochondria in BPH-1, AR-BPH-1, sh-NC-AR-BPH-1 and sh-MMP7-AR-BPH-1 cells. The corresponding statistical analysis is presented in statistical graph on the right. **F** Immunofluorescence analysis of mt-Keima in BPH-1, AR-BPH-1, sh-NC-AR-BPH-1 and sh-MMP7-AR-BPH-1 cells. Scale bar: 10 μm. The statistical analysis is presented on the right. **G** Representative immunofluorescence staining was employed to visualize the co-localization of lysosomes and mitochondria. DAPI (blue) shows nuclear staining; Cy3-immunofluorescence (red) shows mitochondrion；Alexa fluor 488 (Green) shows lysosomes. The scale bars are 20 μm. The corresponding statistical analysis are presented on the right. *p < 0.05, ** *p* < 0.01, *** *p* < 0.001. All aforementioned experiments were independently replicated a minimum of 3 times to ensure reproducibility and data reliability. The data are presented as the means ± SDs. Statistical significance was determined by two-tailed unpaired Student’s t-test **G** or determined by one-way ANOVA with Tukey’s multiple comparisons **C, D, E, F**.

To further evaluate the regulatory effect of MMP7 on mitophagy, autophagosome marker protein LC3B was detected in BPH-1 and anoikis resistance-BPH-1 cells. Compared with ordinary BPH-1 cells, LC3B protein aggregation is more pronounced in anoikis resistance-BPH-1 cells. Furthermore, immunofluorescence staining revealed that MMP7 knockdown significantly reduces LC3B aggregation in anoikis resistance-BPH-1 cells. (Fig. 4A, C). We further examined the co-localization of Parkin (a mitophagy-related key protein) and mito-tracker in BPH-1 and anoikis resistance-BPH-1 cells using immunofluorescence staining. The results revealed that, compared with normal BPH-1 cells, anoikis resistance-BPH-1 cells exhibited significantly enhanced co-localization of Parkin and mito-tracker, whereas MMP7 knockdown markedly reduced this co-localization in anoikis resistance-BPH-1 cells (Fig. 4B, D). Additionally, Parkin was subjected to quantitative subcellular protein analysis, which manifested as an elevated distribution in mitochondrial membranes within anoikis resistance-BPH-1 cells compared with controls, whereas MMP7 depletion markedly reduced Parkin accumulation on mitochondrial membranes, with more expression in the cytoplasm (Fig. 4E). Furthermore, we utilized fluorescent mt-Keima in the mitochondria to detect mitophagy activity in BPH-1 and anoikis resistance-BPH-1 cells. Microscopic analysis revealed that the red-to-green fluorescence ratio was significantly higher in anoikis resistance-BPH-1 cells than in normal BPH-1 cells. In contrast, MMP7 knockdown led to a marked reduction in the red-to-green fluorescence ratio in anoikis resistance-BPH-1 cells (Fig. 4F). Corroborating with these findings, immunofluorescence co-localization experiments revealed that MMP7 knockdown significantly attenuated the co-localization signal of lysosomes and mitochondria, indicating impaired mitophagy (Fig. 4G).

### Identification of VDAC1 as the MMP7 counterpart in mitophagy

**Fig. 5:**
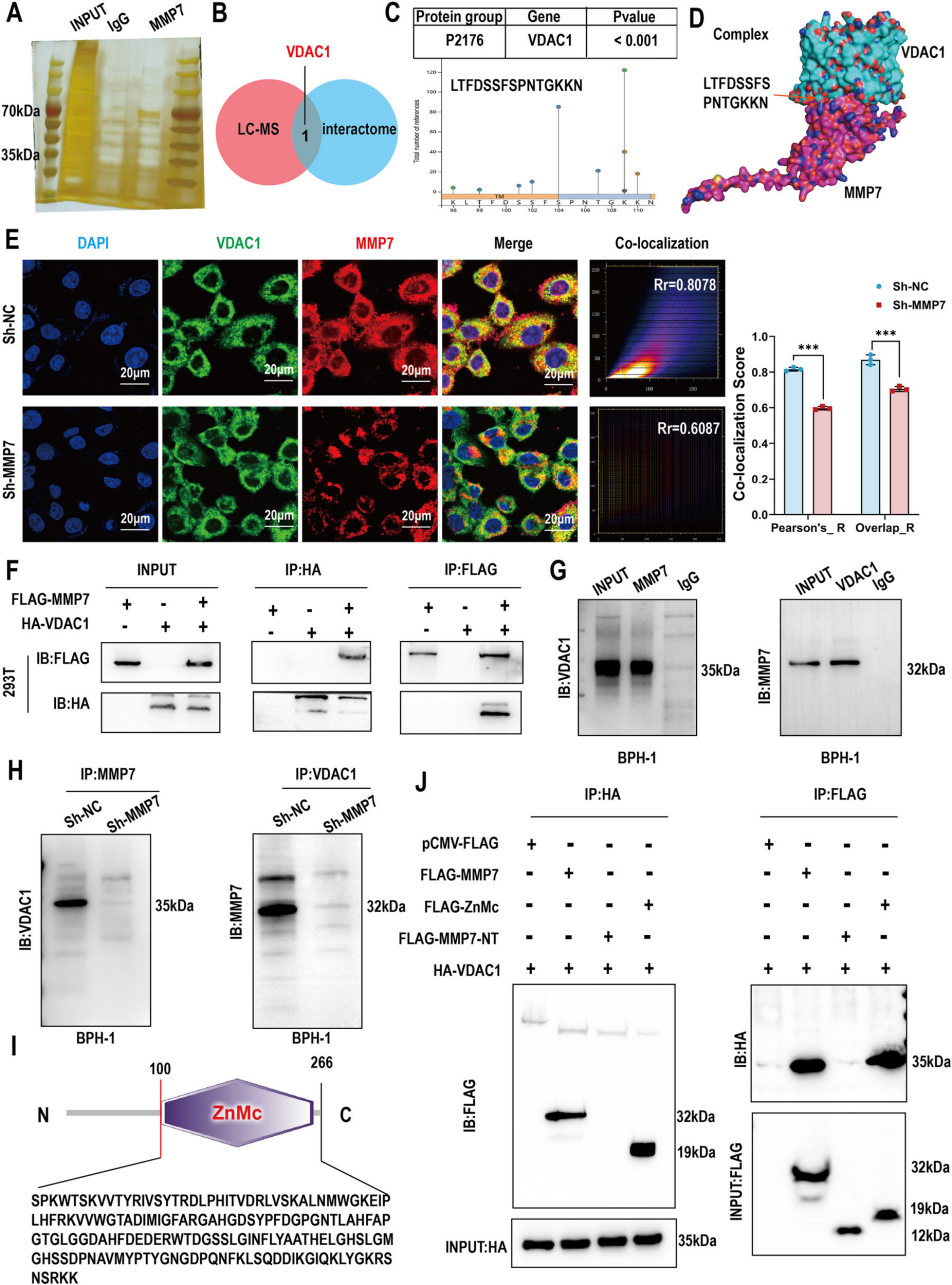
VDAC1 was identified as the counterpart of MMP7 in AR-BPH-1 cells. **A** Representative silver staining images were acquired following immunoprecipitation in AR-BPH-1 cells to identify proteins interacting with MMP7. **B** The number of MMP7-interacting candidate proteins identified by co-IP LC-MS analyses in AR-BPH-1 cells. The data cluster mentioned above intersected with the data cluster from Reactome-mitophagy. VDAC1 was the only candidate protein. **C** Potential interacting amino acid sequences between MMP7 and VDAC1 identified by LC-MS analysis. **D** 3D interaction model between MMP7 and VDAC1 predicted by AlphaFold Server. **E** Representative immunofluorescence staining was employed to visualize the co-localization of VDAC1 and MMP7 in AR-BPH-1 knocked down or not. **F** The co-IP assay was designed ed to detect the exogenous MMP7-VDAC1 interaction in HEK293 T cells with ectopically expressed FLAG-tagged MMP7 and HA-tagged VDAC1. **G** Co-IP assays proved the endogenous MMP7-VDAC1 interaction in AR-BPH-1 cells. **H** The co-IP assay was designed ed to detect the MMP7-VDAC1 interaction in BPH-1 cells with MMP7 knocked down or not. **I** Schematics showing MMP7 contained two protein domains (ZnMc and N-terminal). **J** Co-IP assays proved the interactions between MMP7 and VDAC1 located in the ZnMc domain. *** *p* < 0.001. The data are presented as the means ± SDs. Statistical significance was determined by two-tailed unpaired Student’s t-test **E**.

Quantitative analysis of MMP7-binding proteins was further performed by LC-MS and intersected with a list of mitophagy-related proteins from the Reactome mitophagy database. Notably, voltage-dependent anion channel 1 (VDAC1) emerged as the only overlapped protein (Fig. 5A–C). Subsequently, prediction of the the interaction between MMP7 and VDAC1 in 3D structure by AlphaFold (www.alphafold.com) showed that MMP7 could bind to the β-strand of VDAC1, potentially interfering with VDAC1 oligomerization (Fig. 5D). Moreover, immunofluorescence analysis revealed extensive co-localization of MMP7 and VDAC1 in BPH-1 cells, which was reduced with MMP7 knockdown (Fig. 5E). To further investigate whether MMP7 interacted with VDAC1, we conducted a series of co-immunoprecipitation (co-IP) experiments. As shown in Fig. 5F, plasmids encoding FLAG-tagged MMP7 and HA-tagged VDAC1 were transfected into HEK293T cells, and co-IP analysis revealed an exogenous interaction between MMP7 and VDAC1. Similarly, endogenous MMP7 efficiently interacted with VDAC1 in BPH-1 cells (Fig. 5G). Additionally, the interaction between MMP7 and VDAC1 could be lessened by the loss of MMP7 in BPH-1 cells (Fig. 5H). To verify the specific domains responsible for the association between MMP7 and VDAC1, two truncated mutant plasmids were constructed and transfected into HEK293T cells (Fig. 5I). MMP7 contains an N-terminal propeptide domain (including the “cysteine switch” (PRCGVPD), which maintains the zymogen in an inactive state and prevents self-degradation) and a central catalytic domain (containing a zinc ion and a substrate binding pocket, the active center for hydrolyzing protein substrates, also known as the ZnMc domain). To locate the region within MMP7 that mediates its interaction with VDAC1, two mutants with only the N-terminal domain and the ZnMc domain were constructed. Co-IP analysis revealed that the ZnMc domain of MMP7 was indispensable for its association with VDAC1, while the N-terminal domain of MMP7 was relatively unnecessary for its interaction with VDAC1 (Fig. 5J).

### MMP7 Enhanced polyubiquitination of VDAC1 at K109 and K110

**Fig. 6:**
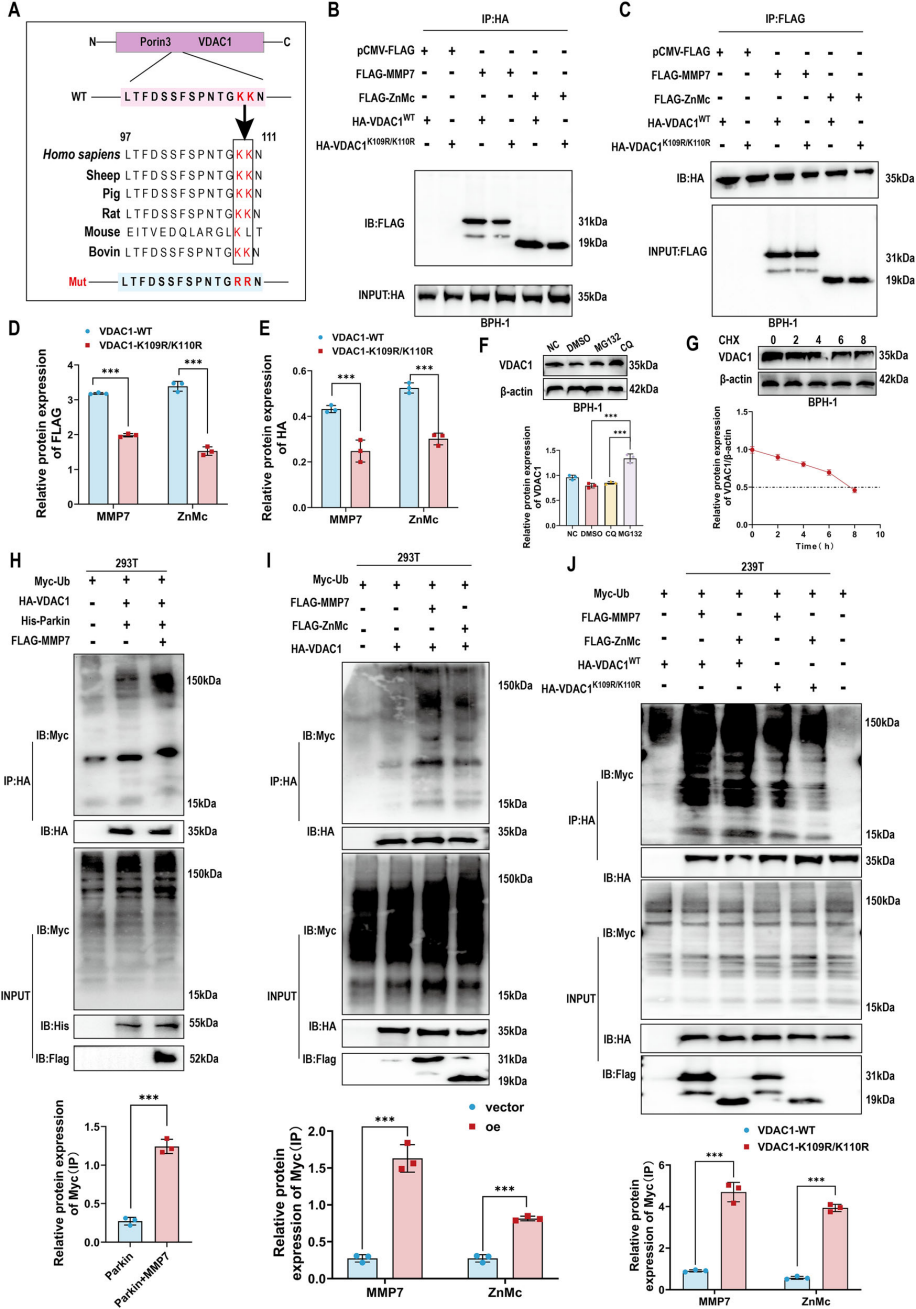
MMP7 enhanced polyubiquitination of VDAC1 at K109 and K110. **A** Conservation analysis for the MMP7-VDAC1 interaction site across different species and schematic of the mutant construct. **B, C** Co-IP assays show the K109/K110 was the key site for the interaction between MMP7 and VDAC1 in BPH-1 cells. **D** Statistical graph of Western blot bands showing the protein expression of FLAG-tagged MMP7 in BPH-1 cells. **E** Statistical graph of Western blot bands showing the protein expression of HA-tagged VDAC1 in BPH-1 cells. **F** BPH-1 cells were treated with DMSO (1 µL), CQ (20 µM) and MG132 (20 µM) for 24 h, followed by collection for Western blot assay. Representative Western blot images show the effect of DMSO, CQ and MG132 on VDAC1 degradation in BPH-1 cells. **G** BPH-1 cells were treated with CHX (50 µg mL−1) for indicated hours, followed by collection for Western blot assay. Representative Western blot images show the half-life of VDAC1 protein degradation. **H** HEK293T cells were transfected with MYC-Ub; MYC-Ub and HA-VDAC1; MYC-Ub, HA-VDAC1 and His-Parkin; MYC-Ub, HA-VDAC1, His-Parkin and FLAG-MMP7 as indicated for 72 h and then treated with 20 µm MG132 for 8 h. Cellular extracts were prepared for IP assays after HA-IP followed by immunoblotting with anti-Myc. The corresponding statistical analysis results are presented below. **I** HEK293T cells were transfected with MYC-Ub; MYC-Ub and HA-VDAC1; MYC-Ub, HA-VDAC1 and FLAG-MMP7; MYC-Ub, HA-VDAC1 and FLAG-ZnMc as indicated for 72 h and then treated with 20 µm MG132 for 8 h. Cellular extracts were prepared for IP assays after HA-IP followed by immunoblotting with anti-Myc. The corresponding statistical analysis results are presented below. **J** HEK293T cells were transfected with MYC-Ub; MYC-Ub and HA-VDAC1wt; MYC-Ub, HA-VDAC1wt and FLAG-MMP7; MYC-Ub, HA-VDAC1wt and FLAG-ZnMc； MYC-Ub, HA-VDAC1K109R/K110R and FLAG-MMP7; MYC-Ub, HA-VDAC1K109R/K110R and FLAG-ZnMc as indicated for 72 h and then treated with 20 µm MG132 for 8 h. Cellular extracts were prepared for IP assays after HA-IP followed by immunoblotting with anti-Myc. The corresponding statistical analysis results are presented below. *** *p* < 0.001. The data are presented as the means ± SDs. Statistical significance was determined by two-tailed unpaired Student’s t-test **D, E, H, I, J** or determined by one-way ANOVA with Tukey’s multiple comparisons **F**.

As demonstrated by LC-MS, the binding sequence of VDAC1 to MMP7 was predicted as “LTFDSSFSPNTGKKN” (Fig. 5C). Notably, lysine residues K109 and K110 within this sequence have been identified as polyubiquitination sites of VDAC1. Conserved domain analysis showed that these residues were evolutionarily conserved across species, indicating their potential functional importance in mediating the interaction between VDAC1 and MMP7 (Fig. 6A). Furthermore, VDAC1 mutants were constructed with two conserved lysines (K109/K110) replaced by arginine (R109/R110), and co-IP experiments were performed in HEK293T cells co-expressed FLAG tagged MMP7 with HA tagged wild-type VDAC1 (VDAC^WT^) or K109R/K110R mutant VDAC1 (VDAC1^K109R/K110R^). Compared with VDAC^WT^, VDAC1^K109R/K110R^ showed reduced binding capacity to full-length MMP7 and the ZnMc domain (Fig. 6B–E). We further showed that MG132 (a proteasome inhibitor) effectively restrained the degradation of VDAC1 in BPH-1 cells compared to CQ (an autophagolysosomal inhibitor) (Fig. 6F). Additionally, A cycloheximide (CHX) chase experiment showed that the half-life of VDAC1 was approximately 6-8 h in BPH-1 cells, suggesting that VDAC1 degradation primarily occurred *via* the polyubiquitin-proteasome pathway (Fig. 6G). Moreover, co-IP experiments in HEK293T cells transfected with VDAC1, MMP7 and Parkin confirmed that more polyubiquitination modification of VDAC1 by Parkin was detected in the presence of MMP7 overexpression (Fig. 6H). Moreover, co-IP experiments in HEK293T cells transfected with VDAC1 and MMP7 or its ZnMc domain truncated mutant indicated that the ZnMc domain of MMP7 facilitated VDAC1 polyubiquitination by binding to VDAC1 (Fig. 6I). Further co-IP experiments in HEK293T cells transfected with VDAC^WT^ or VDAC1^K109R/K110R^ together with MMP7 or its ZnMc domain truncated mutant demonstrated the regulatory effect of MMP7 and its ZnMc domain on polyubiquitination of VDAC1^K109R/K110R^ mutant was markedly weaker than that of VDAC^WT^ (Fig. 6J). These findings suggested that the ZnMc domain of MMP7 could promote polyubiquitination modification of VDAC1 by Parkin through targeting K109 and K110 of VDAC1.

### MMP7 Promoted mitophagy by disrupting VDAC1 oligomerization

**Fig. 7:**
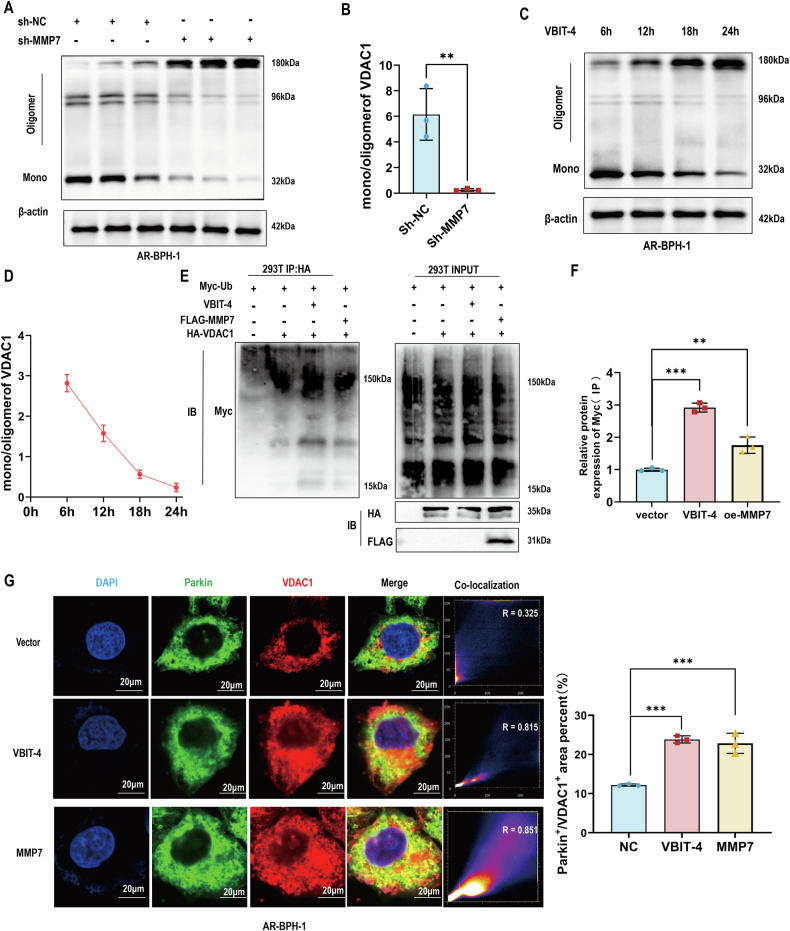
MMP7 promotes mitophagy by interfering with the oligomerization of VDAC1. **A** Oligomer and monomer of VDAC1 protein in AR-BPH-1 cells with MMP7 knockdown. **B** The statistical graph of Western blot bands reveals the relative value of the monomeric to oligomeric forms of the VDAC1 protein in AR-BPH-1 cells with MMP7 knockdown. **C** Oligomer and monomer of VDAC1 protein in BPH-1 cells after 10 μM VBIT-4 agent treatment for 6 h, 12 h, 18 h and 24 h. **D** The statistical graph of Western blot bands showing the relative value of the monomeric to oligomeric forms of the VDAC1 protein in BPH-1 cells after 10 μM VBIT-4 agent treatment for 6 h, 12 h, 18 h and 24 h. **E** HEK293T cells were transfected with MYC-Ub; MYC-Ub and HA-VDAC1; MYC-Ub, HA-VDAC1 and VBIT-4 (10 μM, 24 h); MYC-Ub, HA-VDAC1 and FLAG-MMP7 as indicated for 72 h; and then treated with 20 µm MG132 for 8 h. Cellular extracts were prepared for IP assays after HA-IP followed by immunoblotting with anti-Myc. **F** Statistical graph of Western blot bands showeing the protein expression of Myc (IP: HA) in C). **G** Statistical graph of yellow punctum in AR-BPH-1, AR-BPH-1 treated with VBIT-4 (10 μM) for 24 h and AR-BPH-1 with MMP7 knocked down (*n* = 3). **G** Representative immunofluorescence staining analysis of the co-localization of Parkin and VDAC1 in in AR-BPH-1 cells (vector, VBIT-4 (10 μM) for 24 h and overexpressed MMP7). DAPI (blue) shows nuclear staining; Cy3-immunofluorescence (red) shows VDAC1 protein；Alexa fluor 488 (Green) shows Parkin protein. The scale bars are 20 μm. The corresponding statistical analysis results are presented on right. ** *p* < 0.01, *** *p* < 0.001.The data are presented as the means ± SDs. Statistical significance was determined by two-tailed unpaired Student’s t-test **B** or determined by one-way ANOVA with Tukey’s multiple comparisons **F, G**.

As shown in Fig. 7A, B, knockdown of MMP7 in BPH-1 cells substantially enhanced the formation of VDAC1 oligomers. Similarly, BPH-1 cells treated with VBIT-4 (a VDAC1 oligomerization inhibitor) significantly increased VDAC1 oligomer levels (Fig. 7C, D). Furthermore, co-IP experiments in HEK293T cells ectopically expressing VDAC1 along with MMP7 or VBIT treatment showed that increased ubiquitination of VDAC1 was significantly elevated in the presence of VDAC1 oligomerization inhibition or MMP7 overexpression (Fig. 7E, F). Moreover, confocal microscopy analysis demonstrated that MMP7 overexpression or VBIT-4 treatment both promoted co-localization of VDAC1 and Parkin, manifested by elevation of yellow fluorescence (Fig. 7G). Together, all these findings indicated that MMP7 inhibited VDAC1 oligomerization, resulting in accumulation of VDAC1 monomers, thus generating more anchor sites to recruit Parkin, which mediated polyubiquitination of VDAC1.

### Inhibiting MMP7 suppressed benign prostatic hyperplasia (BPH) progression in vivo

**Fig. 8:**
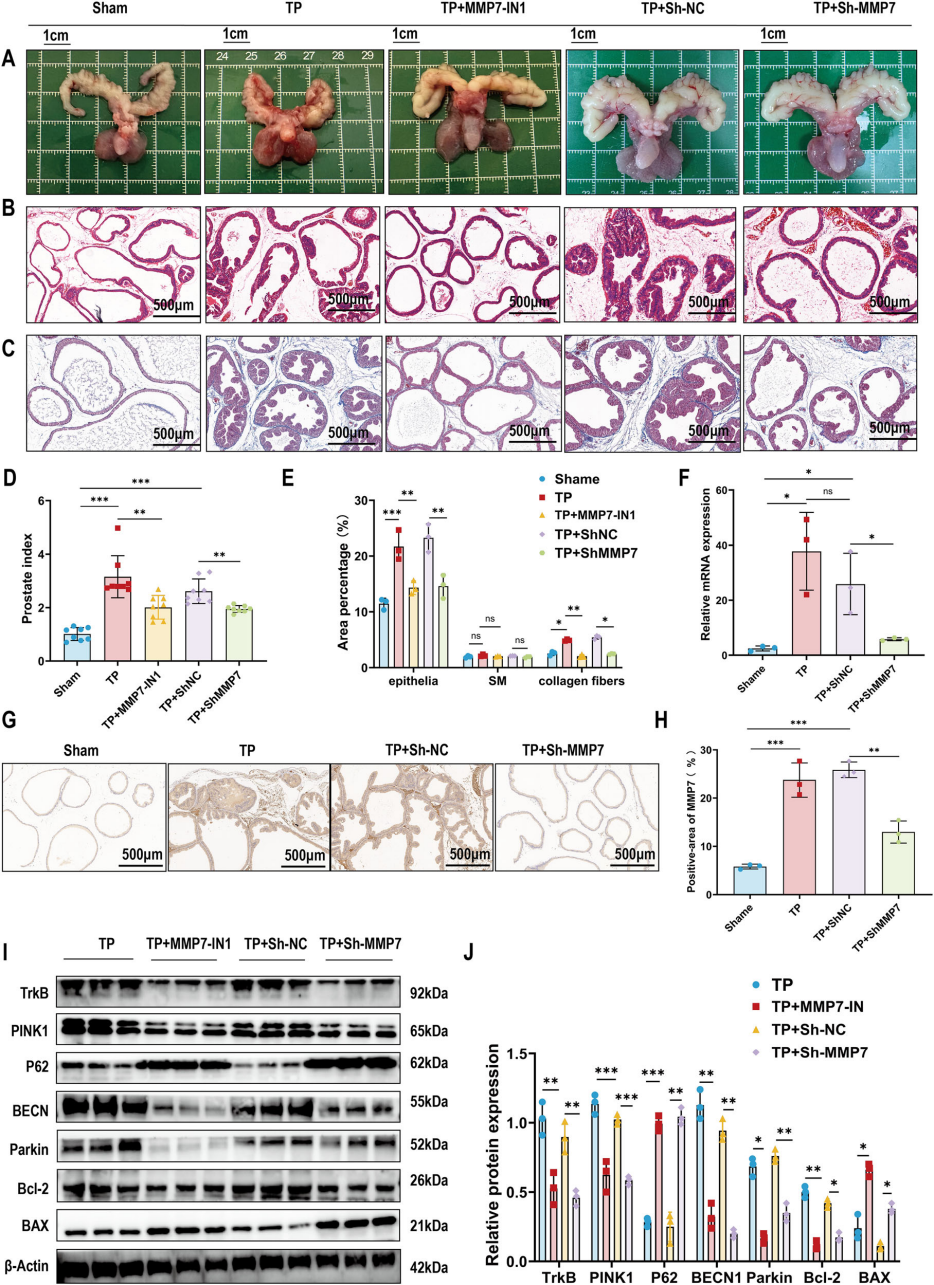
Inhibition of MMP7 suppresses BPH progression by impeding mitophagy in vivo. **A** Representative macroscopic images of the prostate, bladder, and seminal vesicles from male SD rats are presented from left to right as follows: control group (Sham), testosterone propionate (TP) group, TP + MMP7-IN1 group, TP + sh-NC group, and TP + shMMP7 group. Each group contains n = 8 animals. The scale bar is 1 cm. **B** Representative H&E stained images of the prostate, bladder, and seminal vesicles from male SD rats are presented from left to right as follows: control group (Sham), testosterone propionate (TP) group, TP + MMP7-IN1 group, TP + sh-NC group, and TP + shMMP7 group. Each group contains *n* = 8 animals. The scale bar is 500 μm. **C** Representative micrographs of Masson’s trichrome-stained prostate tissue sections from male SD rats in the Sham group, testosterone propionate (TP) group, TP + MMP7-IN1 group, TP + sh-NC group, and TP + sh-MMP7 group (*n* = 8). In these images, prostatic epithelial cells are stained brownish red, smooth muscle (SM) cells are stained red, and collagen fibers are stained blue. The scale bar corresponds to 500 μm. **D** Statistical graphical representation of prostate index in male rats across different treatment groups (*n* = 8 per group). **E** Statistical graphical representation of the area proportions of epithelial components, smooth muscle components, and collagen fibers in Masson’s trichrome-stained prostate tissue sections (*n* = 8 per group). **F** The qRT-PCR technique was used to display the mRNA expression levels of MMP7 in the prostate tissues of representative rats (*n* = 3) in control group (Sham), testosterone propionate (TP) group, TP + sh-NC group, and TP + shMMP7 group. **G** Representative IHC staining images of in-situ MMP7 protein in prostate tissue sections from male SD rats are presented from left to right as follows: control group (Sham), testosterone propionate (TP) group, TP + sh-NC group, and TP + shMMP7 group. Each group contains *n* = 3 prostate. The scale bar is 500 μm. **H** Statistical graph depicting the quantification of MMP7-positive areas measured using Image J software(*n* = 3 per group). **I** Representative Western blot bands showing the expression levels of AR (TrkB), proliferation (Bcl-2), apoptosis (BAX), mitochondrial autophagy (PINK1 and Parkin), and autophagy-related markers (P62 and BECN1) in prostate tissues of rats from the TP group, TP + MMP7-IN1 group, TP + sh-NC group, and TP + sh-MMP7 group. **J** Statistical graph of Western blot bands show the protein expression of TrkB, Bcl-2, BAX, PINK1, Parkin, P62 and BECN1. ns < 0.05, *p < 0.05, ** *p* < 0.01, *** *p* < 0.001. The data are presented as the means ± SDs. Statistical significance was determined by two-tailed unpaired Student’s t-test **J** or determined by one-way ANOVA with Tukey’s multiple comparisons **D, E, F, H**.

We further explored the therapeutic potential of MMP7 in vivo. As shown in Fig. S4, a TP-BPH rat model with predominantly epithelial hyperplasia was established. Compared with the control group, ventral prostate weight and prostate index (prostate weight/body weight) significantly increased in TP-BPH rats. Conversely, both the MMP7-IN1 treatment group and the sh-MMP7 injection group exhibited markedly reduced ventral prostate weight and prostate index compared with the TP-BPH rats (Fig. 8A, D and Table 1). Histologically, H&E staining revealed a relatively increased epithelial component, characterized by enlarged glands lined with tall columnar epithelium in TP-BPH rats. Masson trichrome staining further indicated that TP-induced prostate hyperplasia in rats primarily occurred in the epithelial component, followed by relatively increased deposition of collagen fibers, whereas no significant effect on smooth muscle. Notably, all these alterations in TP-BPH rats were completely reversed upon the treatment of either MMP7-IN1 or sh-MMP7 (Fig. 8B, C, E). Additionally, it was observed that the mRNA and protein levels, as well IHC stain of MMP7, were significantly upregulated in prostate tissues of TP-BPH rats while substantially decreased with sh-MMP7 treatment (Fig. 8F–H). We also examined the protein levels of essential regulators. It was found that the anoikis resistance level, autophagy, and mitophagy in TP-BPH rat prostates were all attenuated with the downregulation of TrkB, BECN1, PINK1, and Parkin along with the upregulation of P62, whereas the apoptosis level was elevated with the upregulation of BAX along with the downregulation of Bcl-2 in the context of MMP7 knockdown *via* MMP7-IN1 or sh-MMP7 (Fig. 8H, I). These results provided strong in vivo evidence that knockdown of MMP7 alleviated BPH progression by reducing mitophagy and anoikis resistance activity.

**Table 1:** The body weight, prostate weight and prostate index of rats in different treatment groups (*n* = 8).

Group	Body weight (g) Initial	Body weight (g) Final	Ventral prostate weight (mg)	Prostate index
Sham	216.6(6.1)	456.078 (35.24)	452.5(84.1)	1.0(0.2)
TP	219.8(9.7)	393.91(35.24)	1232.5(255.4)	3.2(0.8)
TP + MMP7-IN1	221.0(6.4)	426.6(28.66) **	853.7(178.1)	2.0(0.4)
TP+sh-NC	222.4(8.9)	413.11(25.45)	1105.0(157.8)	2.7(0.5)
TP + sh-MMP7	214.0(5.7)	449.11(32.27) ^###^	877.1(113.4)	1.9(0.1)

Note: TP - Testosterone Propionate; MMP7-IN1 - Specific Inhibitor 1 of MMP7; ** indicates that p < 0.01 for TP + MMP7-IN1 versus TP group; ### indicates that *p* < 0.001 for TP + sh-MMP7 versus TP + sh-NC group; Prostate index (mg/g): Prostate weight (mg) / Rat body weight (g) at the time of sampling; Data are presented as mean ± standard deviation.

### Correlation analysis of MMP7 protein expression levels with clinical data of BPH patients

**Fig. 9:**
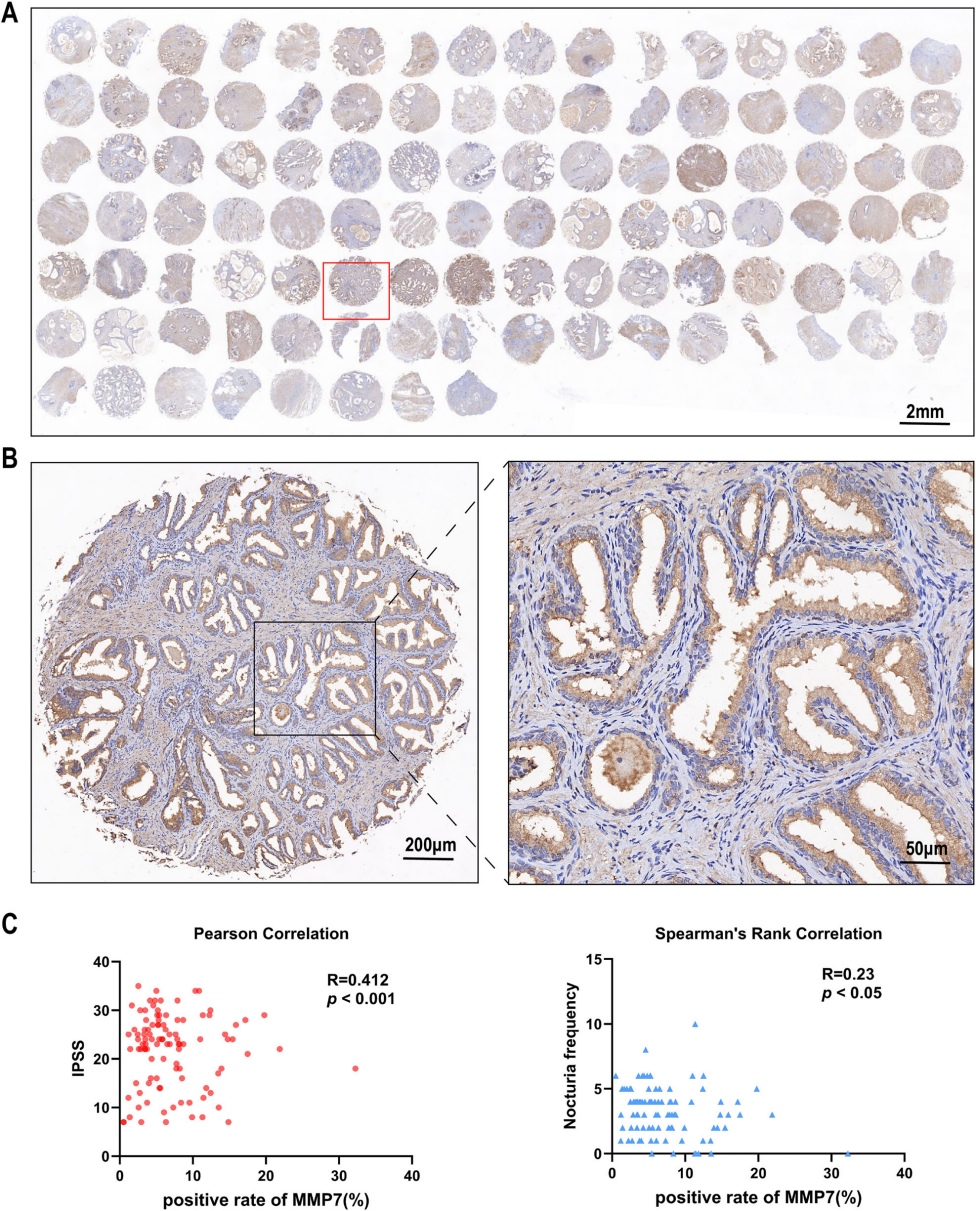
Immunohistochemical staining of MMP7 in tissue microarray of BPH. **A** Immunohistochemical staining images of MMP7 in 104 cases of human hyperplastic prostate tissue microarrays. The scale bar is 2 mm. **B** Magnified views of representative staining patterns are presented. The scale bar is 200 μm, and 50 μm. **C** The correlation analysis between the positive rate of MMP7 protein and the clinical characteristics of the patients showed that the IPSS score and the nocturia frequency were positively correlated with the positive rate of MMP7 protein. The data are presented as the means ± SDs. Statistical significance was determined by Pearson’s correlation coefficient (for IPSS) and Spearman’s correlation coefficient (for Nocturia frequency).

Finally, the correlation between expression of MMP7 and clinical parameters related to BPH was analyzed. As shown in Fig. 9A, B, TMA analysis containing 104 human hyperplastic prostate tissues revealed that MMP7 was highly expressed in the epithelial compartment compared with that of stromal compartment in BPH specimens. Furthermore, the expression level of MMP7 was positively correlated with IPSS (*r* = 0.412, *P* < 0.001) and nocturia frequency (N, *r* = 0.231, *P* < 0.05) in BPH patients (Fig. 9C and Table 2). These findings indicated that elevated MMP7 expression in hyperplastic prostate tissues was associated with the more severe the lower urinary tract symptoms in patients were.

**Table 2:** Correlation analysis of clinical information and MMP7 expression level in BPH patients.

	Correlation Coefficient	*p*-Value	statistical method
Age	−0.06981	0.481	Spearman
BMI	0.05622	0.571	Pearson
PV	−0.1240	0.210	Pearson
tPSA	−0.09493	0.360	Pearson
fPSA	−0.1071	0.302	Pearson
Qmax	−0.01223	0.943	Pearson
RUV	−0.1483	0.315	Pearson
IPSS	0.4124	<0.001^***^	Pearson
N	0.2309	0.022^*^	Spearman

*BMI* body mass index, *PV* prostate volume, *tPSA* total prostate specific antigen, *fPSA* free prostate specific antigen, *IPSS* international prostate symptom score, *Qmax* maximum flow rate, *RU* residual urine, *N* Nocturia. * *p* < 0.05.

## Discussion

Recent studies have shed light on the abnormal regulation of PCD in BPH progression, which could be independent of classical hormone signaling. The canonical form of PCD is apoptosis, which is widely acknowledged to be attenuated in the pathogenesis of BPH. Our previous studies have identified that GRP78 could activate the AKT/mTOR pathway in BPH, promoting prostate cell proliferation and inhibiting apoptosis [35]. In addition to apoptosis, a novel form of PCD, ferroptosis, has recently been explored in the etiopathogenesis of BPH in our lab. We observed that GPX3 induced mitochondrial apoptosis through suppressing the AMPK/ERK1/2 pathway, while it attenuated autophagy-related ferroptosis *via* AMPK/mTOR in BPH [10]. Anoikis, a distinct form of PCD, plays an essential role in tissue development (facilitates the acini formation of prostate) and homeostasis [36]. Under pathological conditions, detached cells can resist anoikis and survival, a phenomenon termed anoikis resistance.

Interestingly, the unique “braking” phenomenon observed in prostate epithelial cells could be closely associated with anoikis resistance, involving overlapping signaling pathways and molecular components [21, 22]. To identify whether anoikis resistance exists in BPH, TrkB was validated as a molecular marker for anoikis resistance by Douma et al [37]. It was found that TrkB effectively inhibited apoptosis of cells detached from ECM but exhibited no anti-apoptotic activity in adherent cells. Also, the expression of TrkB was shown to be positively correlated with anoikis resistance [37, 38]. As expected, our current study demonstrated that TrkB was upregulated in both human and rat hyperplastic prostate, indicating that the anoikis resistance level was elevated in BPH prostate tissues.

To explore the underlying mechanism of anoikis resistance in BPH, we screened out a subpopulation of BPH-1 cell with anoikis resistance characteristics using three-dimensional ultra-low adsorption culture dishes. With this anoikis resistance cell model and human prostate tissues, transcriptome sequence study was performed. MMP7 was ultimately identified as a novel differentially expressed gene involved in anoikis resistance of BPH. As the smallest member of the MMPs family, MMP7 has become a focus molecule in ECM remodeling and disease progression due to its unique role in substrate specificity, tissue distribution characteristics, and multi-dimensional functional capabilities [24, 39, 40]. It has been reported that MMP7 is involved in various cancers and other diseases. In prostate cancer, MMP7 could degrade proteoglycans on the tumor cell surface, then activate signaling pathways including FAK, AKT, and FOXM1, leading to reduced cell cohesion, increased cell motility and promotion of tumor cell metastasis [41]. In colon cancer cells, MMP7 was transported to the nucleus through a nuclear localization signal and shown to directly bind to the survivin promoter to enhance its transcription [42–44]. Our study further demonstrated that both mRNA and protein expression levels of MMP7 were significantly elevated in human hyperplastic prostate tissues, with predominant localization observed in the epithelial compartment. In parallel, bioinformatics analysis of publicly available single-cell RNA sequencing data revealed marked upregulation of MMP7 in epithelial cells derived from BPH tissues, particularly within the luminal and basal epithelial subpopulations. Additionally, it was found that knockdown of MMP7 significantly reduced the anoikis resistance level in BPH-1 cells. Thus, our data suggested that MMP7, instead of just acting as a secretory protease, can also be intracellularly involved in anoikis resistance in BPH under specific conditions.

We further investigated the functional pathway by which MMP7 could regulate anoikis resistance in human prostate epithelial cells. In the anoikic environment, transcriptome sequence data indicated that MMP7 was associated with multiple mitochondrial membrane or mitochondrial function-related genes and enriched in autophagy, particularly mitophagy signaling pathways. Notably, mitophagy is a core mechanism for maintaining cellular energy metabolism homeostasis and mitochondrial quality in various stress triggers, including inflammation, hypoxia, nutrient deficiency, and anoikis [30, 45]. The initiation of mitophagy depends on the PINK1-Parkin-dependent ubiquitination pathway and receptor-mediated non-ubiquitination pathway [31]. When the mitochondrial membrane potential is lost, PINK1, which stably accumulates on the outer mitochondrial membrane, activates the E3 ubiquitin ligase Parkin. The latter ubiquitinates mitochondrial membrane proteins such as VDAC1 and MFN2, and then recruits autophagy receptor proteins to bind to LC3B-labeled autophagosomes [46, 47]. Finally, the PINK1-Parkin-dependent ubiquitination pathway is thereby initiated. Our research reported that MMP7 knockdown reduced the OCR in BPH-1 cells, whereas MMP7 overexpression enhanced it, indicating that MMP7 promoted oxidative metabolism in BPH-1 cells. Furthermore, under anoikis-inducing conditions, MMP7 depletion in anoikis resistance-BPH-1 cells led to a marked decrease in mitochondrial membrane potential (ΔΨm), accompanied by severe mitochondrial structural abnormalities—including fragmentation into short rod-shaped and granular forms, loss of cristae, and outer membrane rupture—suggesting impaired mitochondrial integrity. These findings implied that MMP7 played an important role in preserving mitochondrial morphology and function in anoikis resistance-BPH-1 cells. Notably, we observed elevated levels of mitophagy and pronounced Parkin translocation to mitochondria in anoikis resistance-BPH-1 cells under anoikis-inducing conditions, both of which were attenuated upon MMP7 knockdown, indicating that MMP7 modulates mitophagy in response to anoikis. Therefore, we hypothesized that MMP7 might influence mitophagy through regulating the PINK1-Parkin-dependent ubiquitination pathway.

To delve deeper into the role of MMP7 in modulating mitophagy, we performed proteomic analysis, which indicated a direct interaction between MMP7 and VDAC1 on the mitochondrial membrane. Thus, these findings suggested that MMP7 might directly bind to VDAC1 intracellularly to exert its function. Subsequently, our co-IP analyses demonstrated that the C-terminal ZnMc domain of MMP7 directly binds to VDAC1, and mutations in the conserved sites (K109 and K110) of VDAC1 could partially disrupt the interaction between VDAC1 and MMP7. A number of studies have demonstrated that VDAC1 is localized to the outer mitochondrial membrane (OMM), where it regulates metabolic processes and energy exchange, and serves as an important mitochondrial substrate for Parkin, mediating mitophagy and apoptosis in response to mitochondrial damage [48, 49]. Parkin-mediated ubiquitination of VDAC1 predominantly encompasses two distinct forms: monoubiquitination and polyubiquitination [50, 51]. Monoubiquitination of VDAC1 could modulate apoptosis through regulating mitochondrial calcium uptake [52–55]. When Parkin induces polyubiquitination of VDAC1, the ubiquitinated VDAC1 initiates Parkin-mediated mitophagy through the recruitment of the p62/sequestosome 1 (SQSTM1) and LC3B to mitochondria [49]. Ham et al. demonstrated that the polyubiquitination of VDAC1 plays a key role in PINK1 and Parkin-mediated mitophagy, with the identified polyubiquitination sites located at lysine residues 12, 20, 53, 109, and 110 (Poly-K) [56]. Consistent with their report, we found that MMP7 enhanced Parkin-mediated ployubiquitination of VDAC1, which was markedly reduced in VDAC1 ^K109R/K110R^ mutants. Therefore, we speculated that MMP7 could physically associate with VDAC1 through its ZnMc domain, leading to ubiquitination at K109 and K110 of VDAC1, which in turn would promote a Parkin-mediated process.

Furthermore, we investigated how MMP7, a non-ubiquitin ligase, could regulate the ubiquitination of VDAC1. Previously, Liu et al. demonstrated that glycerophosphocholine phosphodiesterase 1 (GPCPD1) modulated VDAC1 ubiquitination through regulation of its oligomerization [57]. Similarly, He et al. found that ETS proto-oncogene 1 (ETS1) mediated VDAC1 ubiquitination via oligomerization [58]. VDAC1 is a transmembrane β-barrel pore protein that forms various oligomeric forms through interaction with its β-chain components [59, 60]. As the primary channel protein of the OMM, VDAC1 dynamically regulates the opening of the mitochondrial permeability transition pore (mPTP) and the release of cytochrome c through its oligomerization state, which is closely associated with the induction of apoptosis [61, 62]. Additionally, the monomer of VDAC1 can recruit more Parkin than the oligomer, thereby enhancing polyubiquitination [57]. Indeed, our study revealed that MMP7 could disrupt oligomerization of VDAC1, which would increase the level of VDAC1 monomers and enhance Parkin recruitment.

Finally, we translated our in vitro studies into the in vivo environment. To explore the intracellular role of MMP7 protein synthesized in prostate epithelial cells themselves, we selected the targeted inhibitors MMP-IN1 and sh-MMP7 lentivirus. MMP-IN1 was synthesized using a hybrid strategy combining small molecules with peptides, allowing the small molecule and peptide components to interact specifically with the S1’ subsite and the non-catalytic subsite of MMP-7, respectively. This design confers potent inhibitory activity against MMP-7 as well as favorable subtype selectivity [63], while sh-MMP7 lentivirus targets post-transcriptional regulation, simulating the knockdown of MMP7. Wang et al. found that intracapsular administration of a lentiviral vector (Lv-sh-METTL3 and Lv-sh-PTEN) into the rat prostate effectively suppressed the expression of METTL3 and PTEN in prostate tissue [64]. Our data revealed that the TP induced BPH rat model manifests a significant hyperplasia in the epithelial compartment. Compared with the blank control group, the ventral prostate weight and prostate index of the TP-BPH group rats were significantly increased. Inhibiting MMP7 significantly reduced the ventral prostate weight, prostate index, epithelial layer thickness and collagen fiber proportion of hyperplastic rats. Consistently, inhibiting MMP7 can down-regulate the anti-apoptotic protein Bcl-2 and up-regulate the pro-apoptotic factor BAX, suggesting that the mitochondrial-dependent apoptosis pathway of rat prostate cells is activated. On the other hand, the down-regulation of TrkB expression in the prostates of rats with MMP7 interference indicated that anoikis was increased. In addition, in our current study, MMP7 depletion could reduce the elevated autophagic activity in hyperplastic prostates, which led to a decrease in the expression of the autophagy-related protein BECN1 and an increase in the expression of P62. Our previous studies also found that autophagy-related proteins (LC3B and BECN1) were up-regulated in human hyperplastic prostate tissue and TP-treated rat prostate tissue [10]. Meanwhile, inhibiting MMP7 lessened the expression of mitophagy-related proteins PINK1 and Parkin, indicating that mitophagy in rat prostates were attenuated.

Additionally, we analyzed the correlation between MMP7 expression and clinical data of BPH patients and found that the expression of MMP7 was positively correlated with IPSS score and nocturia frequency. BPH was characterized by a decrease in epithelial and stromal cells apoptosis, which resulted in the enlargement of prostate volume, resulting in compression of the urethra [65]. The progression of BPH leads to the aggravation of storage and voiding symptoms in patients, along with a higher IPSS score. This suggests that MMP7 might exacerbate tissue hyperplasia and functional disorders and be closely related to the severity of patient symptoms. Thus, it could be a potential diagnostic marker and a therapeutic target.

Our study identified that aberrant mitophagy regulated by the MMP7-VDAC1 axis is a key mechanism underlying anoikis resistance induced by stromal-epithelial microenvironmental imbalance in BPH tissues. This finding provides novel translational insights for the precision diagnosis and treatment of clinical BPH. For instance, MMP7 and TrkB can act as potential targets for novel targeted pharmacotherapy against BPH, laying an experimental foundation for the development of new intervention strategies for BPH patients with resistance to current first-line drugs. Clinically, a subset of BPH patients exhibit resistance to first-line therapeutic agents such as alpha-blockers and 5α-reductase inhibitors, and the dysregulation of the prostatic tissue microenvironment coupled with enhanced survival capacity of hyperplastic cells represents a crucial underlying mechanism for such drug resistance [66, 67]. Inhibition of aberrantly activated mitophagy can reverse anoikis resistance in BPH cells and ameliorate stromal-epithelial microenvironmental imbalance, which may restore the sensitivity of drug-resistant cells to first-line drugs. Furthermore, the specific modulation of mitophagic activity is expected to enhance the therapeutic efficacy of first-line drugs on BPH tissues by remodeling the prostatic tissue microenvironment [68]. Additionally, the expression levels of MMP7, TrkB, and mitophagy-related proteins may serve as potential molecular biomarkers for clinically evaluating disease progression and the sensitivity to first-line drug therapy in BPH patients, thus providing a reference for the implementation of personalized management of BPH. However, the regulatory role of mitophagy has only been verified in cellular and animal models in the present study. Whether mitophagy intervention can reverse drug resistance in clinical BPH samples remains to be further investigated via large-scale clinical cohort studies and in vivo targeted intervention experiments, which will validate the clinical application value of mitophagy modulation for reversing drug resistance in BPH. Meanwhile, the potential crosstalk between mitophagy and the signaling pathways of first-line BPH therapeutic agents warrants in-depth elucidation in subsequent research.

Overall, our novel data revealed that MMP7 could promote the recruitment of Parkin by disrupting the oligomerization of VDAC1, thereby enhancing Parkin-mediated polyubiquitination of VDAC1. This led to the activation of mitophagy in prostate epithelial cells, enabling them to resist anoikis and ultimately contributing to the progression of BPH. Indeed, inhibition of MMP7 may represent a promising therapeutic target for BPH.

## Conclusion

**Fig. 10:**
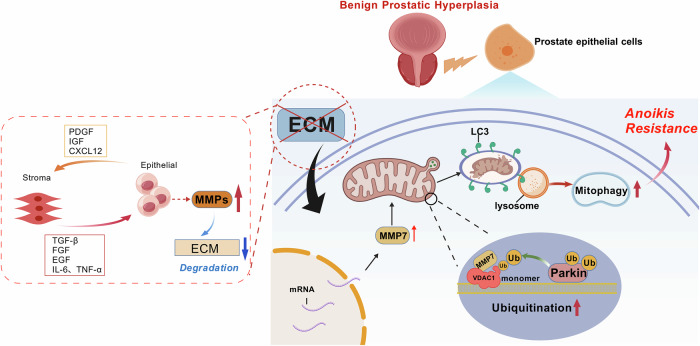
A schematic presentation of the role of MMP7 in AR-induced mitophagy. Dysregulation of epithelial–stromal signaling crosstalk in the prostate tissue leads to upregulated MMP7 expression in epithelial cells, which in turn promotes extracellular matrix (ECM) degradation and disrupts epithelial adhesion to both the ECM and neighboring stromal cells.MMP7 interacts with VDAC1 on the outer mitochondrial membrane, thereby inhibiting VDAC1 oligomerization and increasing the pool of VDAC1 monomers. This facilitates enhanced recruitment of Parkin, leading to Parkin-mediated polyubiquitination of VDAC1, which subsequently triggers mitophagy and enables epithelial cells to resist anoikis.

Our novel data have verified that anoikis resistance is present in BPH. Through the analysis of RNA-seq data from multiple BPH and anoikis resistance-related cells and human prostate tissues, the core gene MMP7 related to anoikis resistance was identified. MMP7 could directly bind to VDAC1 and, by impeding the oligomerization of VDAC1, thus enhance Parkin-mediated polyubiquitination. This process was indispensable for regulating mitophagy and influencing anoikis resistance in BPH. Consequently, MMP7 could act as a modulator of mitophagy, affecting anoikis resistance and thereby determining the occurrence and progression of BPH (Fig. 10).

### Experimental section

Cell line acquisition and culture, flow cytometry analysis, CCK-8 assay, total RNA extraction, RNA reverse transcription, qRT-PCR analysis, total protein extraction, Western blot analysis, immunofluorescence staining, immunohistochemical (IHC) staining, hematoxylin and eosin (H&E) staining and Masson trichrome staining analysis were performed as we previously described [7, 10, 35]. Primer sequences were summarized in Table S1. A list of the antibodies and compounds is included in Tables S2, S3 and S4 respectively.

The sample size was pre-determined to ensure a statistical power of ≥80% (α = 0.05) for detecting the pre-specified effect size (the expected significant differences in mitophagy, anoikis resistance, and stromal-epithelial microenvironment-related indices between BPH experimental and control groups). For in vitro cell experiments, the sample size (biological replicates = 3, technical replicates = 3 per biological replicate) was determined based on preliminary pilot experiments that verified this replicate number was sufficient to detect the above pre-specified effect sizes with minimal variability. For in vivo animal experiments, 8 rats were assigned to each group for BPH in vivo experiments, a sample size determined by preliminary pilot experiments and consistent with the standard sample size in published TP-BPH model studies [10]. This number effectively reduces individual rat variability and adheres to experimental animal 3 R principles.

A rigorous randomization strategy was adopted for the allocation of experimental animals and cell samples to experimental groups prior to all treatments, to avoid systematic bias caused by non-random grouping. Specifically, for SPF-grade male rats with successfully established BPH models, the random number table method was implemented: all eligible rats were numbered sequentially, and corresponding random numbers were obtained from a standard random number table; rats were then randomly assigned to each experimental group (blank control group, BPH model group, positive drug group, experimental group, 8 rats per group) according to the random number distribution, ensuring no significant differences in initial body weight and physiological status among groups (*P* > 0.05). For in vitro cell experiments, cell suspensions with uniform density were randomly allocated to different wells of culture plates (ultra-low attachment/standard culture dishes) for subsequent experimental treatments, to eliminate the well position effect of culture plates.

### Inclusion and exclusion criteria for in vitro cell experiments and in vivo animal studies

Cell/tissue samples inclusion criteria: prostate tissue samples without hemolysis/pollution, RNA/protein extraction purity qualified (A260/A280 = 1.8–2.0); cells in logarithmic growth phase, viability ≥ 90%, no mycoplasma/bacterial contamination. Exclusion criteria: sample contamination, substandard purity of extracted nucleic acids/proteins, low cell viability (<90%), samples with failed experimental treatment (transfection/induction).

Animal studies inclusion criteria: SPF-grade male rats, weighing 200–250 g (replace with your actual weight), without congenital physiological defects/diseases, and successfully constructed BPH model after modeling. Exclusion criteria: accidental death during the experiment, failed modeling, failed prostate tissue sampling/sample contamination, severe infection or other non-experiment-related lesions during the experiment.

### Human normal and hyperplastic prostate tissues and clinical information of BPH patients

Normal human prostate tissue specimens were collected, all from brain-dead male organ donors, with an average age of 20.35 ± 4.20 years. The specimens were collected at the Transplant Center of the Liver and Biliary Institute of Zhongnan Hospital, Wuhan University. All prostate specimens were examined by histopathology and no BPH or other prostate diseases were found. Human hyperplastic prostate tissue specimens were collected, all from BPH patients who underwent transurethral plasmakinetic resection of the prostate. The average age of these patients was 73.38 ± 5.53 years. The specimens were collected at the Department of Urology, Zhongnan Hospital, Wuhan University. Postoperative histopathological examination confirmed all cases as BPH without other prostate diseases, independently confirmed by at least two pathologists.

We simultaneously collected clinical information of 104 BPH patients, including age, body mass index (BMI), prostate volume (PV), total prostate specific antigen (tPSA), free prostate specific antigen (fPSA), maximum flow rate (Qmax), residual urine volume (RUV), international prostate symptom score (IPSS), and frequency of nocturia. The study was conducted in accordance with the principles of the Declaration of Helsinki. The collection and processing of all human specimens and the collection of patient clinical information were approved by the Medical Ethics Committee of Zhongnan Hospital, Wuhan University, and written consent was obtained from the patients or their legal guardians or immediate family members. The study also conformed to the guidelines approved by the Ethics Committee of Zhongnan Hospital, Wuhan University (No. 2021038).

### Bulk RNA-Seq and single-cell transcriptome analysis

Raw gene expression datasets, including GSE119195 and GSE132714, were obtained from the Gene Expression Omnibus (GEO) database (http://www.ncbi.nlm.nih.gov/geo/). Specifically, the GSE119195 dataset includes prostate tissue samples from 5 patients with BPH and 3 individuals with normal prostate tissues [69]. The GSE132714 dataset comprises tissues from 18 BPH patients and 4 histologically confirmed normal controls. Data processing and analysis were performed using R software (version 4.4.3) along with relevant bioinformatics packages [70]. The expression matrices from GSE119195 and GSE132714 were normalized and subsequently merged into a unified dataset. Differential expression analysis was conducted using the R package “limma” (version 3.58.1), with differentially expressed genes (DEGs) identified based on the criteria of FDR < 0.05 and |log2 fold change | > 1. The same “limma” package was employed to assess the expression levels of matrix metalloproteinases (MMPs), with a specific focus on MMP7 in both normal and hyperplastic prostate tissues. Additionally, single-cell transcriptomic data from GSE172357, along with associated annotation files, were downloaded from the CZ CELLxGENE Discover platform (https://cellxgene.cziscience.com/). The R package “Seurat” (version 4.4) was applied to examine the expression pattern of MMP7 across distinct cell populations.

### RNA sequencing

BPH-1 cells were maintained for 7 days on either standard culture dishes or ultra-low adsorption culture dishes. Additionally, BPH-1 cells were transfected with shNC or sh-MMP7 constructs for a duration of 48 h. Total RNA was isolated using TRIzol reagent (Invitrogen, USA), and the concentration and purity of the extracted RNA were determined using the NanoDrop One system (Thermo Scientific, USA). RNA integrity was further verified using the Agilent 2100 Bioanalyzer (Agilent Technologies, USA). Subsequently, RNA-seq libraries were prepared using the VAHTS Universal V6 RNA-seq Library Prep Kit following the manufacturer’s protocol. Transcriptome sequencing and bioinformatics analysis were carried out by Cosmos Wisdom Biotech Co., Ltd. (Hangzhou, China). Differential gene expression, along with GO and KEGG pathway enrichment analyses, were conducted using the Cosmos Wisdom Cloud bioinformatics platform (http://cloud.cwmda.com).

### siRNA, shRNA, and plasmids

siMMP7, siHSPA1A, and siJUN were synthesized by GenePharma (Shanghai, China), and their detailed information is presented in Table S5. For BPH-1 cells, as depicted in Table S6, we altogether designed three types of lentiviral particles carrying targeting sequences. The plasmids of human MMP7-FLAG-tagged, Parkin-FLAG-tagged, VDAC1-HA-tagged, and UB-Myc-tagged were purchased from Miaoling Biology Co., Wuhan, China. The plasmids of human MMP7-NT-tagged (MMP7-N-terminal domain mutant), MMP7-ZnMc-FLAG-tagged (MMP7-ZnMc domain mutant), and MMP7-FLAG-tagged (K109R/K110R) were constructed in our laboratory via standard subcloning.

### The construction and immunohistochemical analysis of TMA

The clinical parameters of 104 patients with were listed in Supplementary Table 8, and descriptive statistics of the clinical parameters were presented in Table 1. Tissue specimens from each of the 104 patient cases were fixed, fabricated into donor wax block sections, and stained with hematoxylin and eosin (H&E) for pathological evaluation and localization of lesion tissues. These evaluations were executed and affirmed by seasoned pathologists. A core with a diameter of 1.5 mm was extracted from each wax block sample. Subsequently, tissue cores from all BPH samples were incorporated into a tissue microarray (TMA), which was then serially sectioned to a thickness of 4 μm. In brief, paraffin sections underwent deparaffinization and were subjected to citrate buffer (pH 6.0) for antigen retrieval, followed by inactivation of endogenous peroxidase activity using 0.3% H_2_O_2_. The sections were then incubated with the pertinent primary and secondary antibodies as detailed in Supplementary Tables S2 and S3, respectively. Antibody localization was visualized through the addition of peroxidase along with 3,3′-diaminobenzidine tetrahydrochloride substrate. Stained sections were imaged using an Olympus DP72 light microscope (Olympus, Japan). Two pathologists who were blinded to the sample types quantified the expression levels of MMP7 in prostate tissues derived from the TMA. Image Pro Plus software (version 6.0) was employed to compute the integral optical density (IOD) sum for MMP7 protein.

$${SUM}=\frac{{IOD}}{{Area}}$$

### Cell transfections

Plasmids and siRNAs were transfected with Lipofectamine 3000 (L3000015, Invitrogen). For plasmids, transfection was conducted when cells achieved 70–90% confluence in 6 - well plates. In tube 1, the Lipofectamine 3000 reagent was diluted with 100 µL of Opti - MEM medium. The plasmids were diluted with 100 µL of Opti - MEM medium and the P3000 reagent was added in tube 2. The diluted plasmids were added to each tube of the diluted Lipofectamine 3000 reagent, thoroughly mixed, and incubated at room temperature (RT) for 15 min. The DNA - lipid complex was added to the cells and the cells were incubated at 37 °C for 2–3 days. While for siRNA transfection of cells, the protocol described for plasmids was followed, but the P3000 reagent was not added when diluting the siRNA, and transfection was performed when cells reached 40–50% confluence in 6 - well plates.

The lentivirus with a titer of MOI = 20 was mixed with 5 μL of the enhanced transfection reagent P3000 in 125 μL of serum-free medium. Simultaneously, 5 μL of Lipofectamine 3000 liposome was pre-diluted in 125 μL of serum-free medium and allowed to stand at room temperature for 5 min. Subsequently, the lentivirus-P3000 mixture and the diluted liposome were mixed dropwise at a 1:1 volume ratio and incubated at room temperature for 15 min to form a stable transfection complex. Then, it was evenly added to the 6-well plate and the culture was continued for 48 h. Subsequently, 2 μg/mL puromycin was added for screening for 7 days. The inhibition efficiency of MMP7 protein expression was verified by Western blotting. The lentivirus with the highest inhibition efficiency was selected for subsequent experiments.

### Establishment of anoikis-resistant BPH cell line

The RWPE-1 cell line, BPH-1 cell line and WPMY-1 were all purchased from Wuhan Procell Life Science & Technology Co., Ltd. (Wuhan, China). Prior to experimental use, these cell lines were recently authenticated via short tandem repeat (STR) profiling and tested negative for mycoplasma contamination.

Cells in the logarithmic growth phase (with a viable cell number of approximately 1×10⁷) were seeded into 10 cm ultra-low attachment culture dishes (Cat. No. 12331, Labselect, China) for suspension culture. The culture medium was refreshed every 48 h following this specific protocol: the entire cell suspension was harvested and centrifuged at 500 × g for 5 min; the resulting cell pellet was gently resuspended in fresh complete medium with careful avoidance of cell clumping, then transferred back to the original ultra-low attachment dishes for continued culture. The cells were cultured under this suspension condition for a total of 7 days, after which the cell suspension was collected and centrifuged at 1000 × g for 5 min. The obtained cell pellet was resown into standard 10 cm cell culture dishes (Cat. No. 704202, Nest, China) for adherent culture. After 24 h of culture, non-adherent cells were gently washed off with phosphate-buffered saline (PBS), and the remaining adherent cells were designated as the anoikis-resistant cells required for subsequent experiments.

After screening AR-BPH-1 cells under anoikis conditions, subsequent assays were performed following standard cell experimental protocols. AR-BPH-1 cells proliferated slightly faster than parental BPH-1 cells and were routinely used 2–3 days after passage, with cell seeding density and specific detection time strictly in accordance with the standard procedure for each corresponding assay. To prevent potential attenuation of anoikis resistance induced by cell passage, all experiments were conducted within 1–2 passages post AR-BPH-1 cell screening. All the aforementioned details have been incorporated into the relevant sections of the manuscript to ensure the clarity and rigor of the experimental design and result interpretation.

### RNA Isolation and quantitative reverse transcription-PCR (qRT-PCR)

These manipulations were performed following our previous works [35]. Briefly, the collected human and rat prostate specimens were rinsed with normal saline to remove residual blood. Aliquots of the specimens were immediately placed in liquid nitrogen and subsequently stored at -80 °C until use. For RNA extraction, the intact frozen tissue samples were retrieved and ground into a fine powder using a cryogenic grinder, after which the subsequent experimental procedures were performed. Total RNA was extracted using the HiPure Total RNA Plus Kit (Cat. No. R411103, Magen, China). The RNA quality and concentration were assessed with a NanoDrop spectrophotometer. First-strand cDNA was synthesized from the total RNA via reverse transcription using the ABScript III RT Master Mix for qPCR with gDNA Remover (Cat. No. RK20429, Abclonal, China). Quantitative real-time PCR was carried out on the StepOnePlus Real-Time PCR System (Thermo Fisher Scientific, USA) using the 2X Universal SYBR Green Fast qPCR Mix (Cat. No. RK21203, Abclonal, China). The specific primer sequences used in this study are listed in Table S1 of the Supporting Information.

### Western blot

These manipulations were performed following our previous works [35]. Human and rat prostate tissue specimens were retrieved from the -80 °C refrigerator and ground into a fine powder using a cryogenic grinder for subsequent experimental procedures. Cells were harvested in accordance with standard cell culture protocols. Tissue powder and cultured cells were lysed on ice for 30 min with a lysis buffer consisting of RIPA buffer, 1 mM PMSF and phosphatase inhibitors, and the resulting supernatants were collected by centrifugation. The whole supernatants were then boiled with 5× SDS-PAGE loading buffer at a volume ratio of 4:1 at 100 °C for 5 min. Total proteins were separated via SDS-PAGE electrophoresis, followed by electrotransfer onto polyvinylidene difluoride (PVDF) membranes. Non-specific binding sites on the membranes were blocked with 5% bovine serum albumin (BSA) in TBST buffer (10 mM Tris-HCl, 150 mM NaCl, 0.5% Tween 20) at room temperature. The PVDF membranes were then incubated with the primary antibodies at 4 °C overnight. After thorough washing, the membranes were incubated with secondary antibodies diluted in 2% BSA at room temperature (RT) for 2 h. Finally, protein bands were visualized by enhanced chemiluminescence (ECL) using the Ultra-Sensitive ECL Detection Kit (Cat. No. PMK0448, Bioprimacy, China) with a chemiluminescence and gel imaging system (BioSpectrum 515 Imaging System, UVP). Information regarding the antibodies utilized in the experiments is provided in Table S2 and S3. Full and uncropped blot images are presented in *Original Western blot bands* of Supplementary Material.

### Real-time mitochondrial oxygen consumption rate (OCR) test

The mitochondrial oxidative phosphorylation function was evaluated through continuous pharmacological intervention, and the OCR was dynamically monitored. Due to the requirements of the reagents and experimental platform, all cells in this part of the experiment are required to be adherently cultured and assayed in specific 96-well culture plates. BPH-1 cells to be measured were inoculated in XF 96-well plates (50,000 cells per well) and allowed to grow overnight at 37 °C. Subsequently, the medium was replaced with XF assay medium (Agilent, 103,575 -100) containing 10 mM glucose, 1 mM pyruvate (Agilent, 103,577 -100 and 103,578 -100), and 2 mM glutamine (Agilent, 103,579 -100) for 1 h at 37 °C. OCR was determined using the Seahorse XF mitochondrial stress test (Agilent). Briefly, the injection ports were loaded to achieve the following final working concentrations in BPH-1 cells: oligomycin (0.5 μM; Agilent, 103,015 -100), FCCP (1 μM; Agilent, 103,015 -100), rotenone (1 μM; Agilent, 103,015 -100), and antimycin A (1 μM; Agilent, 103,015 -100). Data were analyzed using Wave software, and basal respiration (the minimum rate measurement after rotenone+ antimycin A injection) and spare respiratory capacity (the maximum rate measurement after FCCP injection minus non-mitochondrial respiration rate) were calculated.

### Mitochondrial membrane potential detection (JC-1 fluorescence probe method)

BPH-1 cells in the logarithmic growth phase (with a density range of 1×10⁵ - 6×10⁵ cells/ml) were collected and centrifuged for 5 min at 300 xg. The cell pellet was harvested. The cells were resuspended in complete medium to a final volume of 500 μL. The pre-cooled JC-1 staining working solution (Abcam, ab11385) was dropwise mixed with the cell suspension at a 1:1 volume ratio, and gently homogenized for 10 s using a vortex oscillator. Subsequently, the mixture was transferred to a 37°C, 5% CO₂ cell incubator for incubation in the dark for 20 min. After the incubation, the cells were centrifuged at 600 g for 4 min at 4°C, and the supernatant was discarded while retaining the cell pellet. 1 mL of pre-cooled JC-1 staining buffer (10 mM HEPES, 140 mM NaCl, 2.5 mM CaCl₂, pH 7.4) was added to gently resuspend the cells. The centrifugation and washing process was repeated twice to remove unbound probes. Finally, the cells were resuspended in 500 μL of buffer and transferred into a 5 ml centrifuge tube. Subsequent detection was carried out using a flow cytometer in a dark environment.

### Mitochondrial fluorescence staining (Mito-tracker)

An appropriate quantity of BPH-1 cells was taken and centrifuged at room temperature for 5 min at 1000 xg. The supernatant was discarded, and the cells were gently resuspended in the pre-warmed Mito-Tracker Deep Red FM staining (Invitrogen, M22426) working solution at 37°C and incubated for 20–30 min. After the incubation, the cells were centrifuged at room temperature for 5 min at 1000 xg, the supernatant was discarded, and an appropriate volume of the complete culture medium specific for BPH cells was added. The cells were observed under a laser confocal microscope.

### Transmission electron microscope

Cells were fixed for 1 h in 2% paraformaldehyde and 2% glutaraldehyde within 0.1 mol/L phosphate buffer (pH 7.4), followed by post-fixation in 1% OsO4 for 6 h. After undergoing dehydration in graded alcohol solutions (50%, 70%, 80%, 90%, and 100%) for 15 min each time, each sample was embedded in epoxy resin (Sigma-Aldrich, 45,359). The thinned samples (70 nm) were affixed on copper grids (Sigma-Aldrich, TEM-74357), and post-stained with 2% uranyl acetate and 1% lead citrate, dried, and analyzed using a transmission electron microscope (JEOL).

### Mt-Keima

For the mt-Keima detection, the MT-targeted mKeima-Red was procured from Shanghai Hanbio Biotechnology Co., Ltd. The MOI values were set at 3, 10, 30, and 100. The virus stock solution was added to BPH-1 cells and gently mixed. After 4 h of lentiviral infection, the volume was replenished to 100 μL. 24 h after infection, the virus-containing culture medium was aspirated and replaced with fresh complete culture medium, followed by continuous culturing at 37°C. 48 h later, fluorescence was preliminarily observed under a microscope. 72 h after infection, for the group with an infection efficiency of approximately 80% and good cell growth status, the corresponding infection conditions and MOI were considered as the MOI for the subsequent infection experiments. anoikis resistance-BPH-1 cells in ultra-low adsorption 6-well plates were infected. An appropriate amount of virus solution was added to the cell culture dish, which was then sealed. After being placed in a swinging centrifuge, it was centrifuged at a low speed (1200 xg) for 1 h and then placed in the incubator for normal culturing. 72 h after infection, the cells were fixed and mounted, and then observed under a laser confocal microscope (Leica). Visualization of mt-Keima was performed by excitation at 440 nm (for pH = 7) and 550 nm (for pH = 4) with 620 nm emission filters.

### Separation and extraction of cytoplasmic and mitochondrial proteins

Sufficient cells (1×10^7^ cells) were collected and washed twice with pre-chilled PBS at 4°C (pH 7.4, containing 1× protease inhibitor). The cells were centrifuged for 5 min at 800 xg to obtain the cell pellet. The cell pellet was resuspended in 1 mL of pre-chilled cytoplasmic extraction buffer (containing 1× PhosSTOP phosphatase inhibitor and 1 mM DTT), vortexed for 5 s, and then incubated on ice for 15 min. The mixture was centrifuged at 4°C for 5 min at 800 xg. The supernatant was collected and transferred to an ultracentrifugation tube and centrifuged for 30 min at 12,000 xg. The resulting supernatant was the cytoplasmic protein fraction. Subsequently, the pellet after centrifugation at 12,000 xg was resuspended in 100 μL of mitochondrial lysis buffer (containing 1× protease inhibitor and 0.1 mM DTT), vortexed for 10 s, and incubated on ice for 30 min, with vortexing for mixing every 5 min. Subsequently, it was centrifuged at 4°C for 10 min at 13,000 xg, and the supernatant collected was the mitochondrial protein extract.

### Immunoprecipitation-tandem mass spectrometry analysis (IP-MS) and co-immunoprecipitation (Co-IP)

The IP experiment was performed by means of the BeaverBeads Protein A/G Immunoprecipitation Kit (22202 - 100, Beaver). Sufficient amounts of BPH-1 cells (5 ×10^7^ cells) were collected, and lysis buffer (7 M urea, 2 M thiourea, 0.1% CHAPS) was added. Protease inhibitor was added at a ratio of 50:1. The cells were lysed on ice for 30 min and subjected to ultrasonic treatment on ice for 10 min at 30% power with a 3-second interval. After centrifugation at 16,000 xg for 30 min, the supernatant was taken as the total tissue lysate product. The sample concentration was determined by the Bradford method. Protein A/G magnetic beads were pre-incubated and combined with MMP7 primary antibody, FLAG primary antibody, HA primary antibody, or isotype IgG control antibody. Cells were lysed on ice. The supernatant was collected after centrifugation and co-incubated with the antibody-bound magnetic beads overnight. After washing four times, loading buffer was added and boiled for complete denaturation. Western blot and silver staining were used to verify the successful extraction of proteins. Mass spectrometry detection and analysis were accomplished by CWMDA Biotechnology Co., Ltd.

The Co-IP experiments were carried out in accordance with the methods of the aforementioned IP experiments (endogenous IP: 2 × 10^7^ BPH-1 cells; exogenous IP: 1 × 10^6^ HEK 293 T cells), and the corresponding primary antibodies were chosen for the subsequent Western blot analyses.

### Protein stability assay

After treatment with 50 µg/mL CHX for the designated hours, BPH-1 cells were collected for protein extraction and subsequent Western blotting determination. The relative protein level of VDAC1 was calculated using ImageJ software (version 1.54) and normalized to the relative protein level of the loading control.

### Protein ubiquitination assay

Cells were transfected with the corresponding siRNA or plasmid for 48 h. After incubation with 20 µm MG 132 for 8 h, cells were lysed on ice for 30 min using a mixture containing RIPA lysis buffer, 1 mM PMSF, and phosphatase inhibitors. Then, the cell supernatant was incubated overnight with the corresponding antibody, followed by 1-h incubation with magnetic beads. Finally, polyubiquitinated VDAC1 was examined by Western blotting analysis.

### Drug treatment

According to the previous studies of our research group, cells were treated with 10 μM voltage-dependent anion channel 1 oligomerization inhibitor 4 (VBIT-4, TargetMol, China). VBIT-4 was diluted with DMSO. The liquid concentration of each was 100 mM to ensure that the DMSO concentration was less than 0.1% when added to the cells for treatment. In the in vitro experiments, we employed the drug MMP-IN-1. Initially, it was diluted to 10 mM using DMSO, and subsequently, it was further diluted to 2.5 mM with PBS. Then, 50 μl of the final diluted solution was used for intraprostatic injection. For the control group, 50 μl of the solvent (25% DMSO + 75% PBS) was administered.

### Establishment of TP-BPH rat model and drug administration

Establishment of TP-BPH Rat Model: testosterone propionate was uniformly dispersed in refined corn oil at a dose of 5 mg/kg/d by using a high-speed vortex mixer to prepare a 25 mg/mL stock solution. Subcutaneous injections were administered in the neck and back using a 26 G insulin syringe (0.2 mL per rat), and the local area was gently massaged after injection to facilitate absorption. The control group was injected with the same volume of sterilized corn oil, and the injection site was rotated daily to prevent local inflammation.Drug and Lentivirus Injections: A total of 40 six-week-old male Sprague-Dawley (SD) rats, with an initial body weight of 180–200 g, were housed at the Animal Experiment Center of Zhongnan Hospital of Wuhan University for one week and then randomly divided into five groups with eight rats in each group: the control group (Sham), the TP group, the TP + MMP7-IN1 treatment group, the TP + Sh-NC treatment group, and the TP + Sh-MMP7 treatment group (Sh-MMP7 sequences in Table S7). The treatment groups were injected with Testosterone propionat, while the control group was injected with the same volume of sterilized corn oil. Fourteen days after the injection treatment: All rats were anesthetized, a midline incision was made in the lower abdomen to expose the two lateral lobes of the prostate. In the control group (Sham) and the TP group, 50 µL of PBS solution was injected into the prostate using a 31 G needle; in the TP + MMP7-IN1 group, 50 µL of the MMP7-specific inhibitor (MMP7-IN1) was injected; in the TP + Sh-NC group, 50 µL of lentivirus-shNC was injected; and in the TP + Sh-MMP7 group, 50 µL of lentivirus-shMMP7 was injected. The injection sites for all groups were the ventral lobe of the rat prostate.

After an additional 14 days of housing, the rats were weighed, anesthetized with 3% pentobarbital sodium intraperitoneally, and euthanized. The prostate, seminal vesicles, and bladder tissues were obtained and weighed, and molecular biological and histopathological examinations of the ventral lobe of the prostate tissues were performed.

No blinding strategy was implemented, and all experimental procedures were conducted in an open-label (unblinded) manner. This was primarily due to the practical requirements of rat BPH model experiments: core interventions including model establishment, drug administration, and prostate tissue collection required explicit knowledge of group allocation to ensure the accuracy and standardization of operative procedures, and distinct phenotypic differences in prostate tissues among groups precluded effective blinding via sample coding. To minimize potential subjective bias, all experimental operations followed pre-established standardized protocols, and key indices were independently quantified and cross-verified by two researchers.

All animal experimental protocols were approved by the Medical Ethics Committee of Zhongnan Hospital of Wuhan University before being conducted. The experimental protocols underwent comprehensive review and received approval from the Research Ethics Committee and the Institutional Review Board (IRB) (#2021038). All methods were carried out in accordance with relevant guidelines and regulations. The research report conformed to the ARRIVE guidelines to ensure transparency and reproducibility.

### The use of Fiji ImageJ software (Version 2.14.0)

The gray values of Western blot bands, the positive rates of IHC target proteins, and the fluorescence intensities of IF target proteins were quantified and statistically analyzed using Fiji ImageJ software (Version 2.14.0). Notably, IF colocalization was assessed using the “Colocalization Finder” plugin, while mitochondrial length was determined using the “Mitochondrial Analyzer” plugin, both within Fiji ImageJ (Version 2.14.0).

### Statistical analysis

All statistical analyses were conducted through GraphPad Prism (version 10.0) with two-tailed Student’s t-test, one-way or two-way ANOVA followed by Tukey’s multiple comparison test, depending on the situation. Levene’s test was performed to assess the assumption of homogeneity of variances across all experimental groups, and confirmed no statistically significant differences in variance. The data were shown as the mean ± standard deviation (S.D.). *P* < 0.05 indicated a significant difference and *p*-value numbers were shown for each statistical comparison result. For correlation analysis, depending on whether the data are continuous variables, Pearson’s correlation coefficient (for continuous variables) and Spearman’s correlation coefficient (for non-continuous variables) are employed respectively, R > 0.2 and *p* < 0.05 indicated a correlation. The number (n) of tested samples was shown in the figures or legends, where applicable. This study adopted strict bias control measures: all operations followed standardized SOPs, key indicators were independently interpreted and cross-validated by two researchers, original data were double-checked by two people, blind statistical analysis was conducted after removing the grouping labels, and all experiments were completed with at least three biological and technical replicates.

## Supplementary information

Supporting Information

Original Western blot bands

Supporting Information

## Data Availability

The publicly available BPH cohort data were obtained from the GEO database under accession code GSE119195, GSE132714 and GSE145928. The RNA-seq data generated in this study were deposited in the BioProject database under accession code PRJNA1418119. The remaining data are available within the Article, Supporting Information, or Original Data file.
