# Supplementary material for: MMP7-Triggered mitophagy by regulating parkin-mediated ubiquitination of VDAC1 confers anoikis resistance in the epithelium of hyperplastic prostate
Source: Cell Death Dis. 2026 Jun 1;17(1):678. doi: 10.1038/s41419-026-08867-y (PMC13434402; doi:10.1038/s41419-026-08867-y)
Supplement: Supplementary file 1 [file 41419_2026_8867_MOESM1_ESM.pdf]

Supplementary Information

MMP7-Triggered Mitophagy *via* Regulating Parkin-Mediated Ubiquitination of VDAC1 Confered Anoikis Resistance in the Epithelium Hyperplastic Prostate

Figure S1-S4: Pages 2-5
Table S1-S7: Pages 6-12

Figures S1-S4

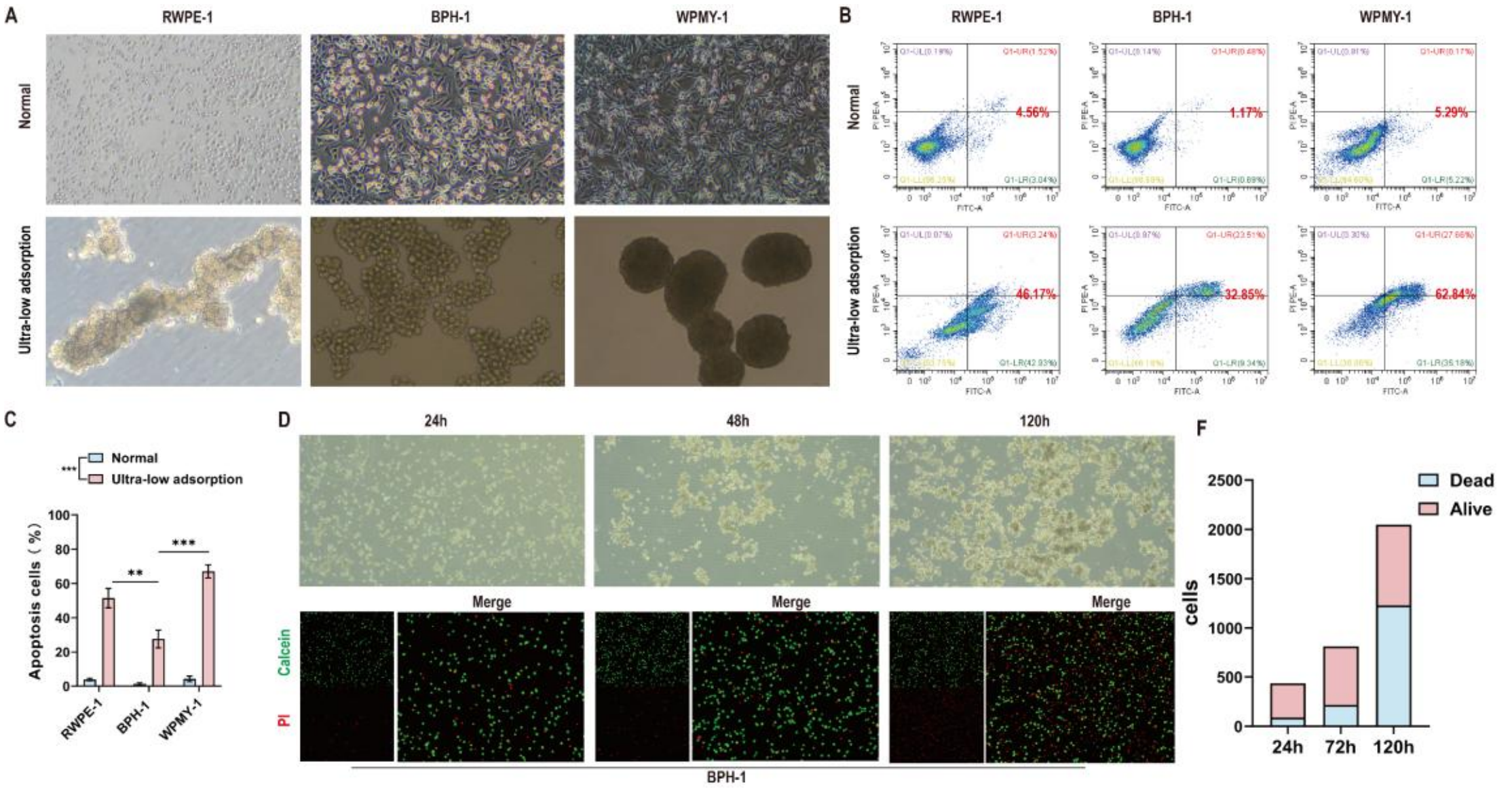

Figure S1. Establishment of AR-BPH-1 Cell Model

(A) The morphological characteristics of RWPE-1, BPH-1, and WPMY-1 cells were examined under bright-field microscopy (10× objective) following 3 days of culture in ultra-low adhesion dishes and normal dishes. **(B)** Apoptosis levels in RWPE-1, BPH-1, and WPMY-1 cells after 3 days of culture in ultra-low adhesion dishes were assessed using flow cytometry, with the lower right (Q4) and upper right quadrants (Q1) indicating the percentages of early and late apoptotic cells, respectively. **(C)** Statistical analysis of apoptosis rates among RWPE-1, BPH-1, and WPMY-1 cells. **(D)** Representative bright-field (upper panel) and live/dead staining (lower panel) images of BPH-1 cells cultured in ultra-low adhesion dishes for 24 h, 72 h, and 120 h. **(E)** Quantitative analysis of live and dead cell ratios in BPH-1 cells cultured under the same conditions for 24 h, 72 h, and 120 h. ** $p < 0.01$ and *** $p < 0.001$.

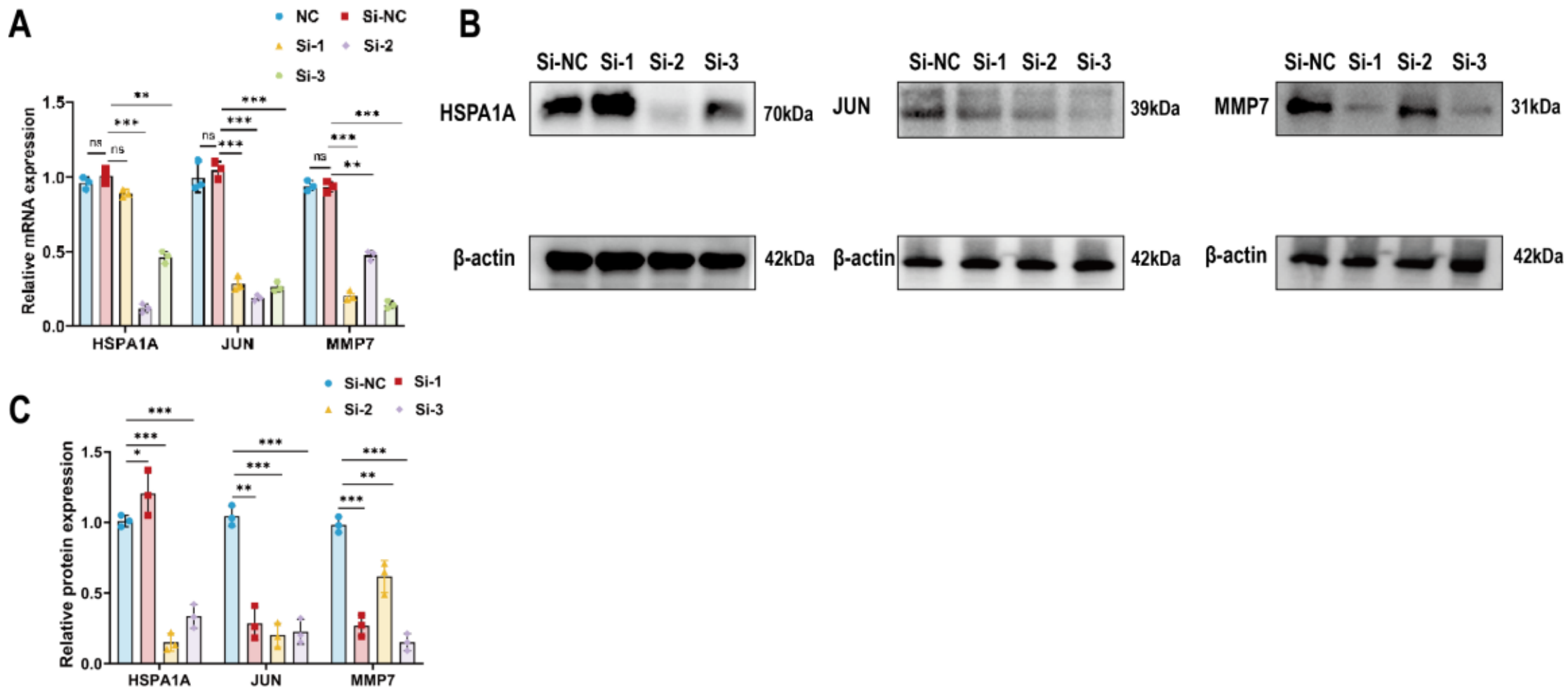

Figure S2 Validation of siRNA Knockdown Efficiency in AR-BPH-1 Cells.

(A) The qRT-PCR analysis of the effects of different siRNAs on the mRNA expression levels of HSPA1A, MMP7, and JUN in BPH-1 cells. **(B)** Representative Western blot bands demonstrating the impact of various siRNAs on the protein expression levels of HSPA1A, MMP7, and JUN in BPH-1 cells. **(C)** The densitometric quantification of the Western blot bands. * $p < 0.05$, ** $p < 0.01$ and *** $p < 0.001$.

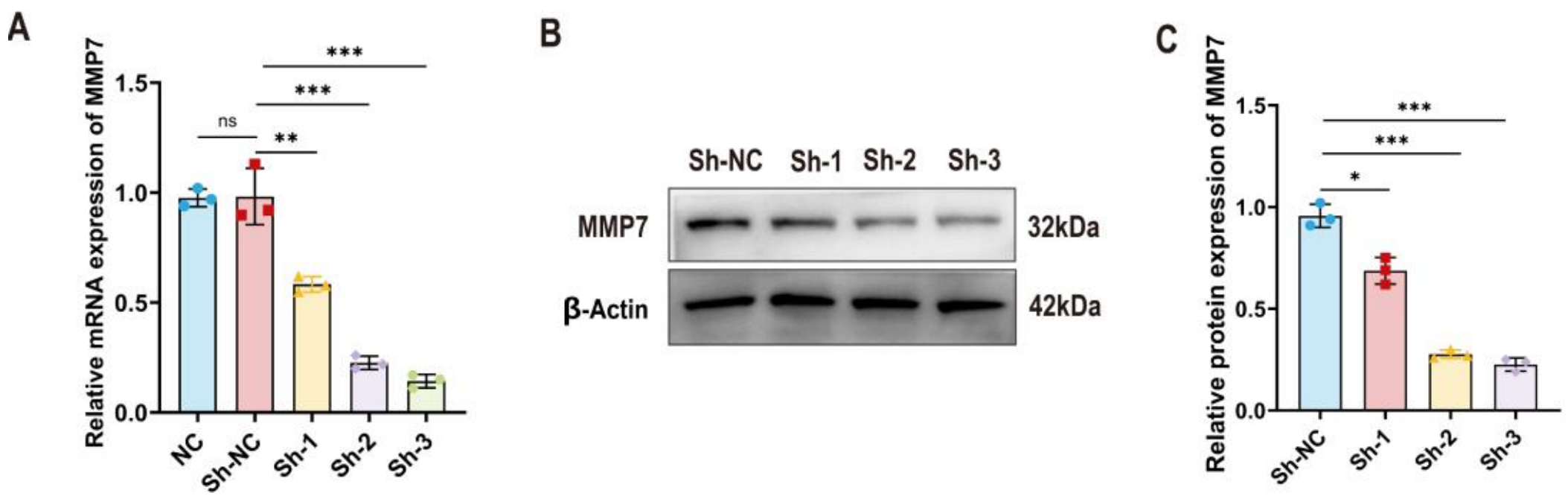

Figure S3 Validation of sh-MMP7 Knockdown Efficiency in AR-BPH-1 Cells.

(A) The qRT-PCR analysis of the effects of individual sh-MMP7 constructs on MMP7 mRNA expression in AR-BPH-1 cells. **(B)** Representative Western blot bands demonstrating the impact of each sh-MMP7 on MMP7 protein expression in AR-BPH-1 cells. **(C)** Densitometric quantification of the Western blot bands. * $p < 0.05$, ** $p < 0.01$ and *** $p < 0.001$.

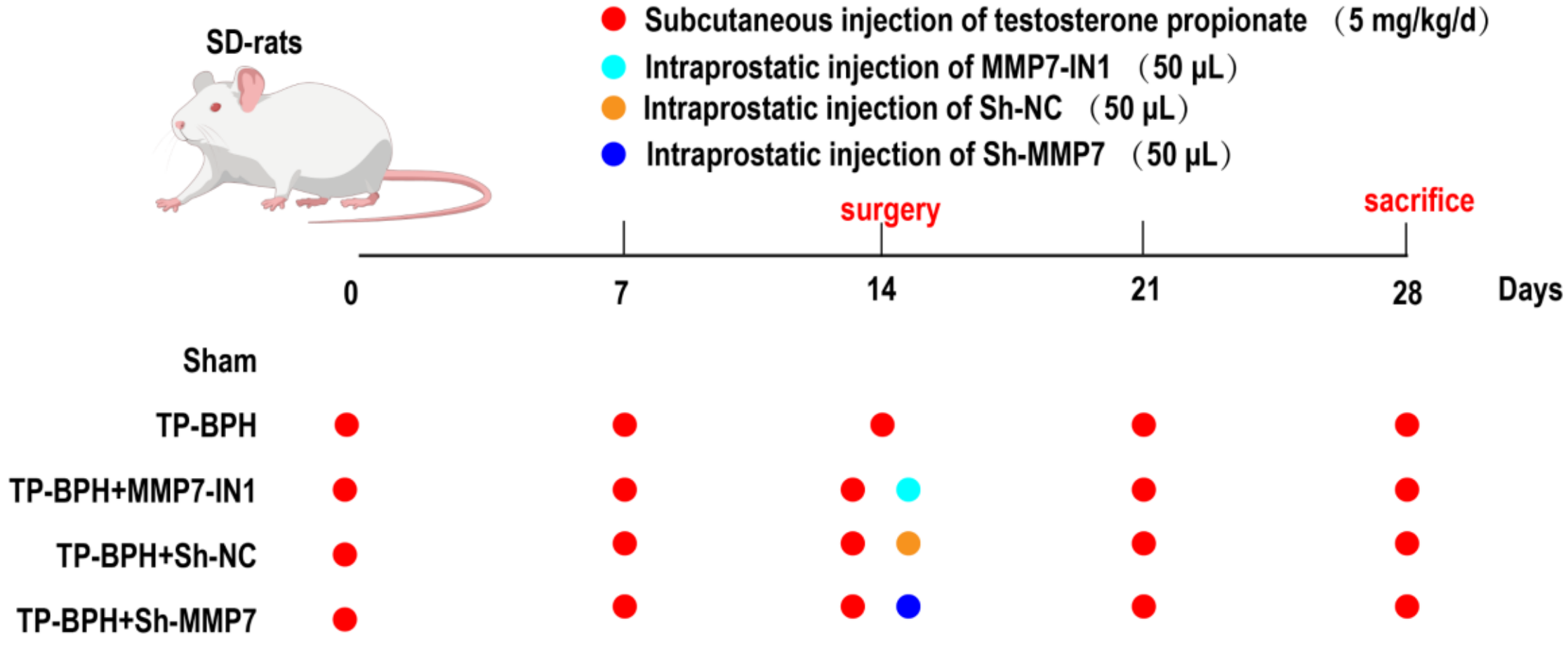

Figure S4. Schematic diagram for evaluating the therapeutic effects of MMP7 inhibition in TP-BPH rats model.

Table S1. Primers used for qRT-PCR assays.

Gene		Primer
HSPA1A	Forward	5′- ACGGTCAAGACCAAGGATGG-3′
HSPA1A	Reverse	5′- AGCGTGTATTTCCGCGTGA -3′
JUN	Forward	5′- TCCAAGTGCCGAAAAAGGAAG -3′
JUN	Reverse	5′- CGAGTTCTGAGCTTTCAAGGT -3′
MMP7	Forward	5′- GAGTGAGCTACAGTGGGAACA -3′
MMP7	Reverse	5′- CTATGACGCGGGAGTTTAACAT -3′
TrkB	Forward	5′- TCGTGGCATTTCCGAGATTGG-3′
TrkB	Reverse	5′- TCGTCAGTTTGTTTCGGGTAAA-3′
β-Actin	Forward	5′- CATGTACGTTGCTATCCAGGC -3′
β-Actin	Reverse	5′- CTCCTTAATGTCACGCACGAT -3′

qRT-PCR: Quantitative reverse transcription PCR.

Table S2. Primary antibodies for Western blot, immunofluorescence and immunohistochemistry

Antibody	Species	Dilution or amount	Source	Cat No.
β-Actin	Rabbit	1:1000(WB)	Proteintech	20536-1-AP
MMP7	Mouse	1:1000(WB); 1:500(IF)<br>1:500(IHC); IP/2 μg	Santa Cruz	sc-515703
TrkB	Rabbit	1:1000(WB); 1:500(IHC)	CST	#4603
α-SMA	Rabbit	1:500(IF)	Proteintech	14395-1-AP
HSPA1A	Rabbit	1:1000(WB)	Proteintech	10995-1-AP
JUN	Rabbit	1:1000(WB)	Proteintech	24909-1-AP
β-Actin	Mouse	1:1000(WB)	Proteintech	66009-1-Ig
Caspase-3	Rabbit	1:1000(WB)	Proteintech	19677-1-AP
PARP-1	Rabbit	1:1000(WB)	Proteintech	13371-1-AP
VDAC1	Mouse	1:1000(WB)；1:500(IF)<br>IP/2 μg	Abcam	sc-390996
TOM20	Rabbit	1:1000(WB); 1:500(IF)	Proteintech	11802-1-AP
Parkin	Rabbit	1:1000(WB)；1:500(IF)	Proteintech	14060-1-AP
α-tublin	Rabbit	1:1000(WB)	Proteintech	14555-1-AP
Flag	Rabbit	1:10000(WB)；IP/1μg	Abmart	R20008
Flag	Mouse	1:5000(WB)；IP/1 μg	Abmart	M20008
HA	Rabbit	1:10000(WB)；IP/1 μg	Proteintech	51064-2-AP
HA	Mouse	1:10000(WB)；IP/1 μg	Proteintech	66006-2-Ig
Myc	Rabbit	1:10000(WB)；IP/1 μg	Proteintech	16286-1-AP
Myc	Mouse	1:10000(WB)；IP/1 μg	Proteintech	60003-2-Ig

IP: Immunoprecipitation;
WB: Western blot;
IF: Immunofluorescence;
IHC: Immunohistochemistry.

Table S3 Secondary antibodies for Western blot, immunofluorescence and immunohistochemistry

Antibody	Species	Dilution or amount	Source	Cat No.
Anti-Rabbit-IgG (H + L)-HRP	Goat	1:10000(WB); 1:100(IHC)	Solarbio	SE134
Anti-Mouse-IgG (H + L)-HRP	Goat	1:10000(WB); 1:100(IHC)	Solarbio	SE131
Anti-Rabbit IgG (H + L), $F(ab')_2$ fragment (Alexa Fluor®488 Conjugate)	Goat	1:50(IF)	CST	4412S
Anti-Rabbit IgG (H + L), $F(ab')_2$ fragment (Alexa Fluor®594 Conjugate)	Mouse	1:50(IF)	CST	4408S

WB: Western blot;
IF: Immunofluorescence;
IHC: Immunohistochemistry.

Table S4. Details of compounds used in the study.

Reagents	Catalog No.	Source
Voltage-dependent anion channel 1 oligomerization inhibitor 4 (VBIT-4)	T13287	TargetMol
MMP-7-IN-1	HY-151540	MedChemExpress

Table S5. The siRNA sequences used in this study.

siRNA		Sequence
si-control	sense	5′-UUCUCCGAACGUGUCAG-3′
si-control	antisense	5′-UGACAUUAAGAUUCAGGGUTT-3′
si-HSPA1A-1	sense	5′-GGCCUUUCCAGGUGAUCAATT-3′
si-HSPA1A-1	antisense	5′-UUGAUCACCUGGAAAGGCCTT-3′
si-HSPA1A-2	sense	5′-GGCAUUCAGAAACUAUAUGTT-3′
si-HSPA1A-2	antisense	5′-AAAGAUGAGCACGUUGCGCTT-3′
si-HSPA1A-3	sense	5′-CCAAGCAGACGCAGAUCUUTT-3′
si-HSPA1A-3	antisense	5′-AAGAUCUGCGUCUGCUUGGTT-3′
si-JUN-1	sense	5′-GCAUCGCUUACAAGGUCAATT-3′
si-JUN-1	antisense	5′-UUGACCUUGUAAGCGAUGCTT-3′
si-JUN-2	sense	5′-CGGCACUUGACAAUGAUCATT-3′
si-JUN-2	antisense	5′-UGAUCAUUGUCAAGUGCCGTT-3′
si-JUN-3	sense	5′-GCACUUCAGAAUGGGAUCUTT-3′
si-JUN-3	antisense	5′-AGAUCCCAUUCUGAAGUGCTT-3′
si-MMP7-1	sense	5′-CUCGAGACUUACCGCAUAUTT-3′
si-MMP7-1	antisense	5′-AUAUGCGGUAAGUCUCGAGTT-3′
si-MMP7-2	sense	5′-GGCAUUCAGAAACUAUAUGTT-3′
si-MMP7-2	antisense	5′-CAUAUAGUUUCUGAAUGCCTT-3′
si-MMP7-3	sense	5′-GCACUGUUCCUCCACUCCATT-3′
si-MMP7-3	antisense	5′-UGGAGUGGAGGAACAGUGCTT-3′

Table S6. The shRNA sequences used in AR-BPH-1 cells.

ShRNA	Sequence（5′-3′）
ShhMMP7-FP-1-GIPZ	TCGAGCAAACTCAAGGAGATGCAATCAAGAG TTGCATCTCCTTGAGTTTG TTTTTTG
shhMMP7-RP-1-GIPZ	GATCCAAAAAACAAACTCAAGGAGATGCAAC TCTTGA TTGCATCTCCTTGAGTTTGC
ShhMMP7-FP-2-GIPZ	TCGAGACCTACAGGATCGTATCATTCAAGAG ATGATACGATCCTGTAGGTTTTTTTG
shhMMP7-RP-2-GIPZ	GATCCAAAAAAACCTACAGGATCGTATCAT CTCTTGA ATGATACGATCCTGTAGGT C
ShhMMP7-FP-3-GIPZ	TCGAG TGCATTTCAGGAAAGTTGTTCAAGA GACAACTTTCCTGAAATGCATTTTTTG
shhMMP7-RP-3-GIPZ	GATCCAAAAAATGCATTTCAGGAAAGTTGT CTCTTGAACAACTTTCCTGAAATGCAC

Table S7 Sequence of Sh-MMP7 lentivirus for animal experiments

ShRNA	**Sequence**（5′-3′）
Sh-MMP7 Top strand	GATCCGCAGTCCTAAGTGGCATTCCAGAACTctcgagAGTTCTGGAATGCCACTTAGGACTGTTTTTTG
Sh-MMP7 Bottom strand	AATTCAAAAAACAGTCCTAAGTGGCATTCCAGAACTctcgagAGTTCTGGAATGCCACTTAGGACTGCG
