# Supplementary material for: MMP7-Triggered mitophagy by regulating parkin-mediated ubiquitination of VDAC1 confers anoikis resistance in the epithelium of hyperplastic prostate
Source: Cell Death Dis. 2026 Jun 1;17(1):678. doi: 10.1038/s41419-026-08867-y (PMC13434402; doi:10.1038/s41419-026-08867-y)
Supplement: Supplementary file 2 [file 41419_2026_8867_MOESM2_ESM.pdf]

Figure 1

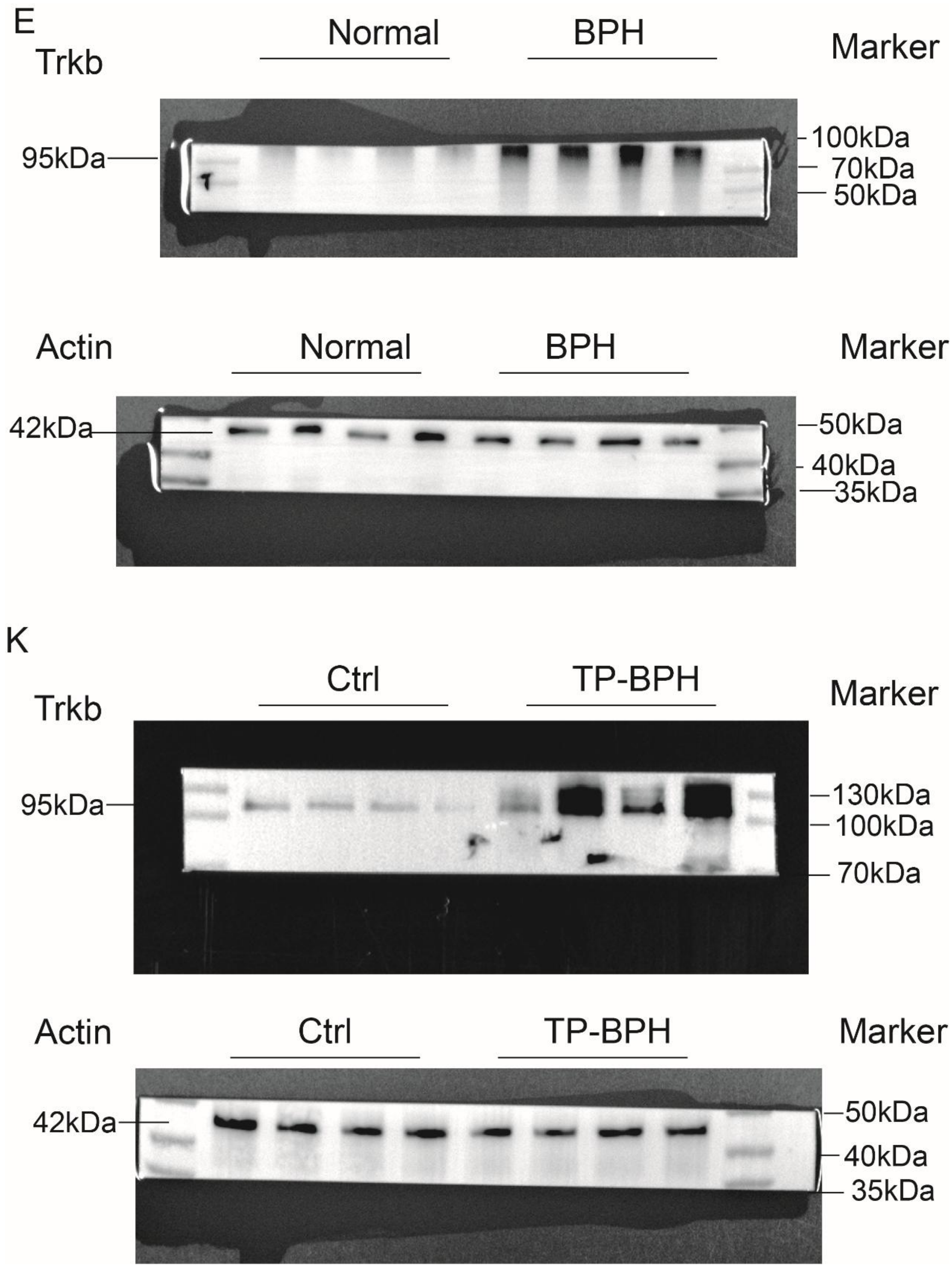
E
Trkb
Normal
BPH
Marker
95kDa
100kDa
70kDa
50kDa
Actin
Normal
BPH
Marker
42kDa
50kDa
40kDa
35kDa
K
Trkb
Ctrl
TP-BPH
Marker
95kDa
130kDa
100kDa
70kDa
Actin
Ctrl
TP-BPH
Marker
42kDa
50kDa
40kDa
35kDa

Figure 2

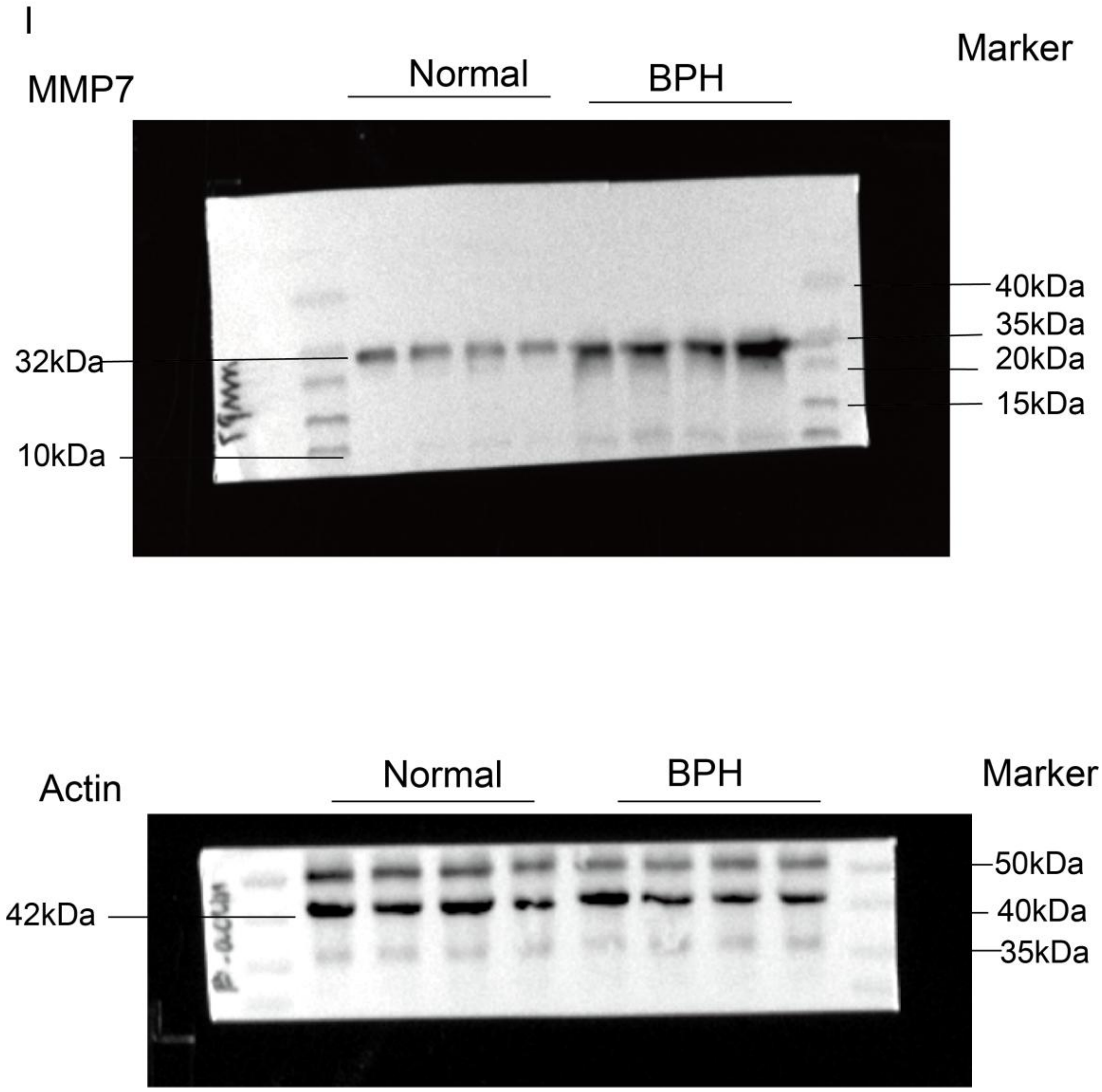
I
MMP7
Normal
BPH
Marker
32kDa
10kDa
40kDa
35kDa
20kDa
15kDa
Actin
Normal
BPH
Marker
42kDa
50kDa
40kDa
35kDa

Figure 3

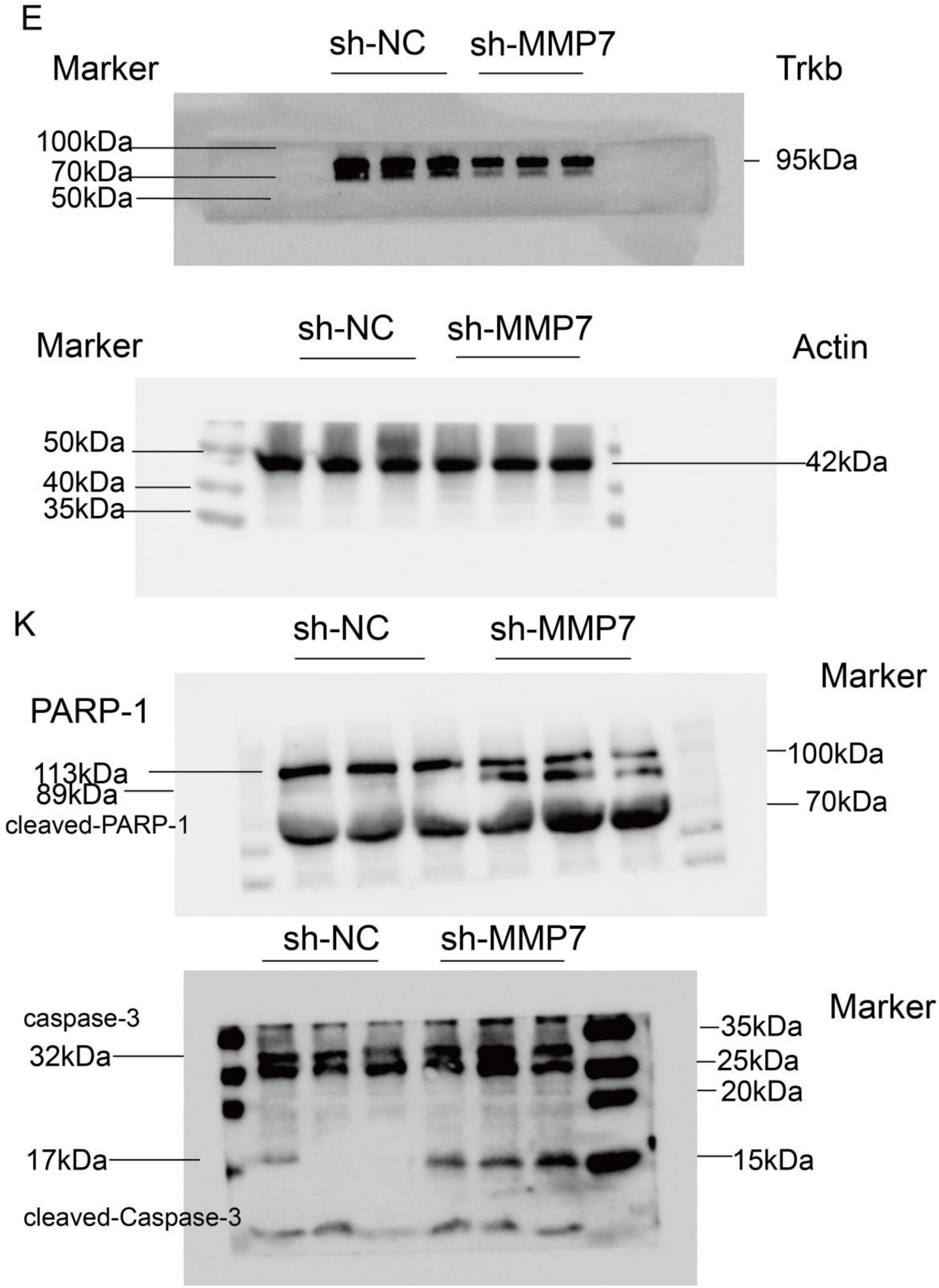

E
Marker
sh-NC
sh-MMP7
Trkb
100kDa
70kDa
50kDa
95kDa
Marker
sh-NC
sh-MMP7
Actin
50kDa
40kDa
35kDa
42kDa
K
sh-NC
sh-MMP7
Marker
PARP-1
113kDa
89kDa
cleaved-PARP-1
100kDa
70kDa
sh-NC
sh-MMP7
Marker
caspase-3
32kDa
17kDa
cleaved-Caspase-3
35kDa
25kDa
20kDa
15kDa

Figure 4

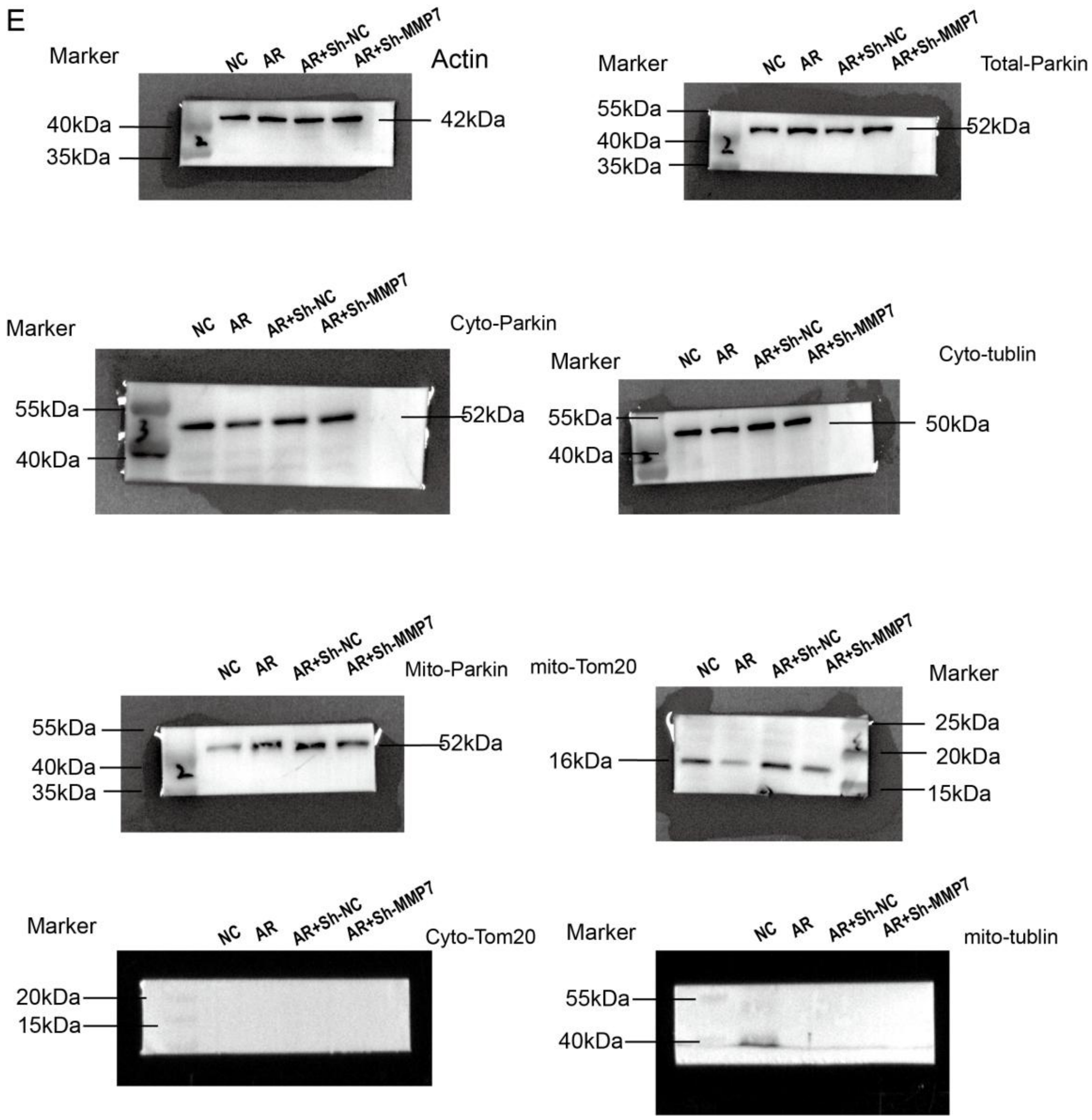
E
Marker
NC
AR
AR+Sh-NC
AR+Sh-MMP7
Actin
40kDa
35kDa
42kDa
Marker
NC
AR
AR+Sh-NC
AR+Sh-MMP7
Total-Parkin
55kDa
40kDa
35kDa
52kDa
Marker
NC
AR
AR+Sh-NC
AR+Sh-MMP7
Cyto-Parkin
55kDa
40kDa
52kDa
Marker
NC
AR
AR+Sh-NC
AR+Sh-MMP7
Cyto-tublin
55kDa
40kDa
50kDa
NC
AR
AR+Sh-NC
AR+Sh-MMP7
Mito-Parkin
55kDa
40kDa
35kDa
52kDa
mito-Tom20
NC
AR
AR+Sh-NC
AR+Sh-MMP7
Marker
16kDa
25kDa
20kDa
15kDa
Marker
NC
AR
AR+Sh-NC
AR+Sh-MMP7
Cyto-Tom20
20kDa
15kDa
Marker
NC
AR
AR+Sh-NC
AR+Sh-MMP7
mito-tublin
55kDa
40kDa

Figure 5

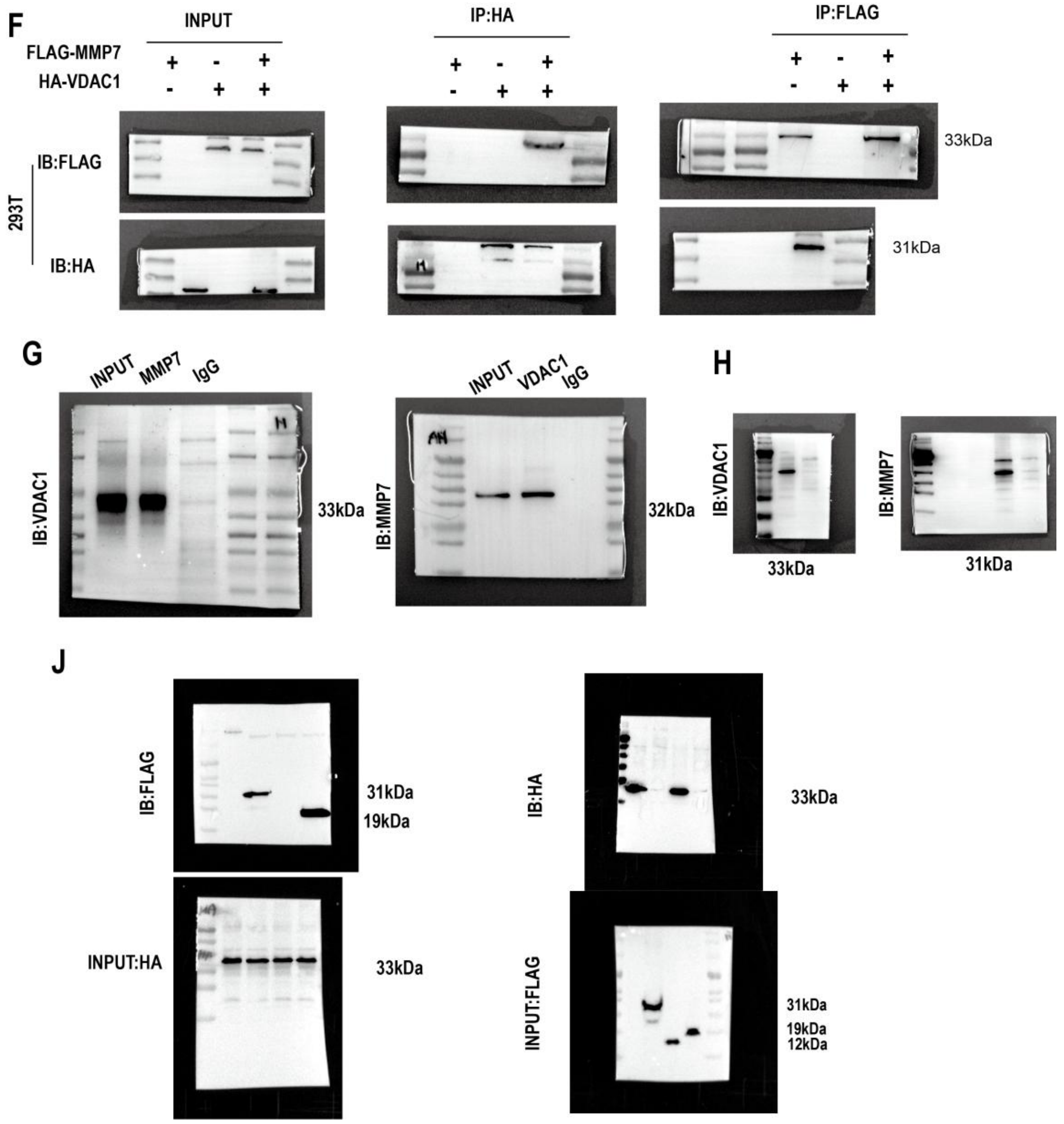

F
INPUT
IP:HA
IP:FLAG
FLAG-MMP7
HA-VDAC1
+ - +
- + +
+ - +
- + +
+ - +
- + +
293T
IB:FLAG
IB:HA
33kDa
31kDa
G
INPUT
MMP7
IgG
IB:VDAC1
33kDa
INPUT
VDAC1
IgG
IB:MMP7
32kDa
H
IB:VDAC1
33kDa
IB:MMP7
31kDa
J
IB:FLAG
31kDa
19kDa
IB:HA
33kDa
INPUT:HA
33kDa
INPUT:FLAG
31kDa
19kDa
12kDa

Figure 6

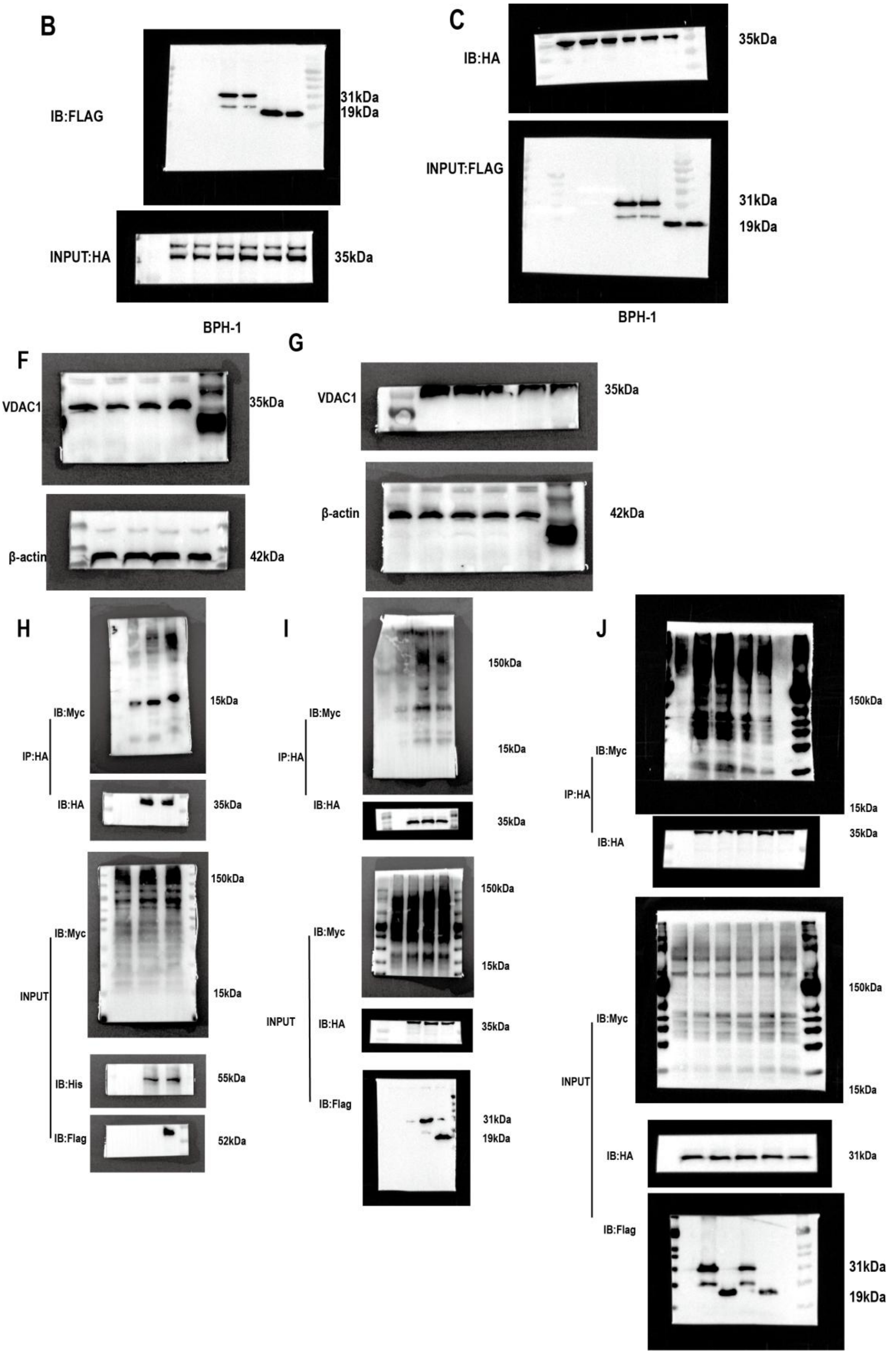
B
IB:FLAG
31kDa
19kDa
INPUT:HA
35kDa
BPH-1
C
IB:HA
35kDa
INPUT:FLAG
31kDa
19kDa
BPH-1
F
VDAC1
35kDa
β-actin
42kDa
G
VDAC1
35kDa
β-actin
42kDa
H
IB:Myc
15kDa
IP:HA
IB:HA
35kDa
150kDa
IB:Myc
INPUT
15kDa
IB:His
55kDa
IB:Flag
52kDa
I
150kDa
IB:Myc
IP:HA
15kDa
IB:HA
35kDa
150kDa
IB:Myc
15kDa
INPUT
IB:HA
35kDa
IB:Flag
31kDa
19kDa
J
150kDa
IB:Myc
IP:HA
15kDa
IB:HA
35kDa
150kDa
IB:Myc
INPUT
15kDa
IB:HA
31kDa
IB:Flag
31kDa
19kDa

Figure 7

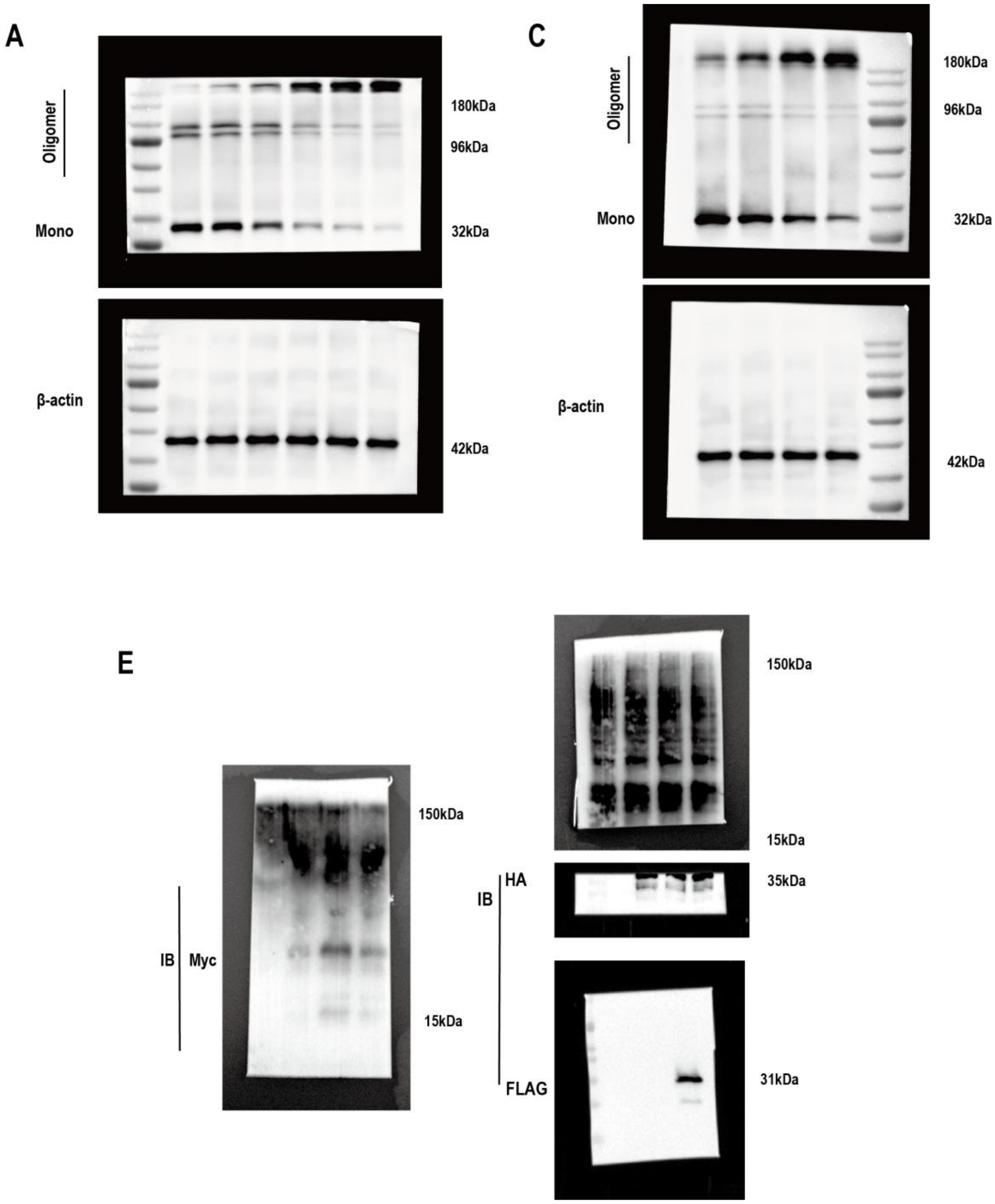
A
Oligomer
Mono
180kDa
96kDa
32kDa
β-actin
42kDa
C
Oligomer
Mono
180kDa
96kDa
32kDa
β-actin
42kDa
E
IB
Myc
150kDa
15kDa
IB
HA
FLAG
150kDa
15kDa
35kDa
31kDa

Figure 8

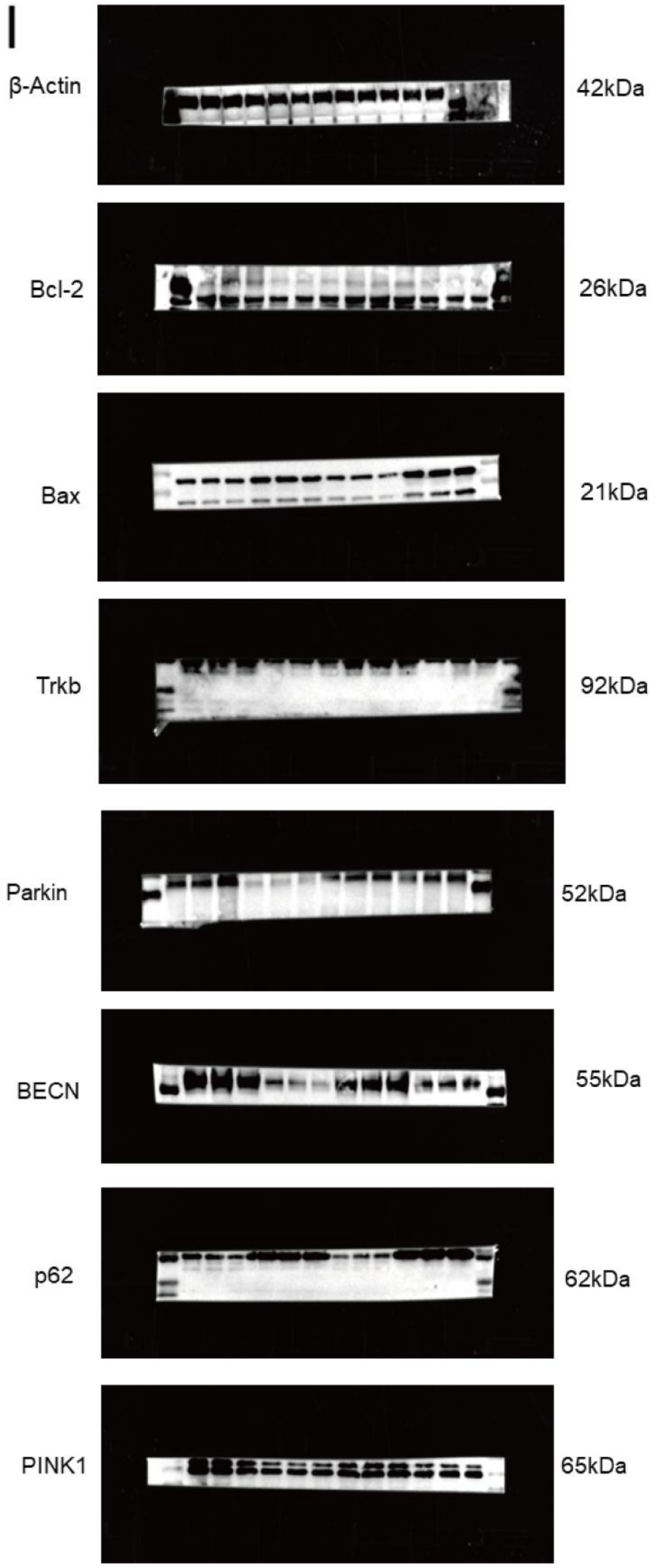

β-Actin
42kDa
Bcl-2
26kDa
Bax
21kDa
Trkb
92kDa
Parkin
52kDa
BECN
55kDa
p62
62kDa
PINK1
65kDa
